# Epidemiology of pediatric surgical needs in low-income countries

**DOI:** 10.1371/journal.pone.0170968

**Published:** 2017-03-03

**Authors:** Elissa K. Butler, Tu M. Tran, Neeraja Nagarajan, Joseph Canner, Anthony T. Fuller, Adam Kushner, Michael M. Haglund, Emily R. Smith, on behalf of the SOSAS 4 Country Research Group

**Affiliations:** 1 Department of Surgery, University of Washington, Seattle, WA, United States of America; 2 Duke University Global Health Institute, Durham, NC, United States of America; 3 Johns Hopkins University School of Medicine, Baltimore, MD, United States of America; 4 Duke University Division of Global Neurosurgery and Neuroscience, Durham, NC, United States of America; 5 Duke University School of Medicine, Durham, NC, United States of America; 6 Department of Neurosurgery, Duke University Medical Center, Durham, NC, United States of America; Universidade Nova de Lisboa Instituto de Higiene e Medicina Tropical, PORTUGAL

## Abstract

**Objective:**

According to recent estimates, at least 11% of the total global burden of disease is attributable to surgically-treatable diseases. In children, the burden is even more striking with up to 85% of children in low-income and middle-income countries (LMIC) having a surgically-treatable condition by age 15. Using population data from four countries, we estimated pediatric surgical needs amongst children residing in LMICs.

**Methods:**

A cluster randomized cross-sectional countrywide household survey (Surgeons OverSeas Assessment of Surgical Need) was done in four countries (Rwanda, Sierra Leone, Nepal and Uganda) and included demographics, a verbal head to toe examination, and questions on access to care. Global estimates regarding surgical need among children were derived from combined data, accounting for country-level clustering.

**Results:**

A total of 13,806 participants were surveyed and 6,361 (46.1%) were children (0–18 years of age) with median age of 8 (Interquartile range [IQR]: 4–13) years. Overall, 19% (1,181/6,361) of children had a surgical need and 62% (738/1,181) of these children had at least one unmet need. Based on these estimates, the number of children living with a surgical need in these four LMICs is estimated at 3.7 million (95% CI: 3.4, 4.0 million). The highest percentage of unmet surgical conditions included head, face, and neck conditions, followed by conditions in the extremities. Over a third of the untreated conditions were masses while the overwhelming majority of treated conditions in all countries were wounds or burns.

**Conclusion:**

Surgery has been elevated as an “indivisible, indispensable part of health care” in LMICs and the newly formed 2015 Sustainable Development Goals are noted as unachievable without the provision of surgical care. Given the large burden of pediatric surgical conditions in LMICs, scale-up of services for children is an essential component to improve pediatric health in LMICs.

## Introduction

The burden of surgical conditions in low-income and middle-income countries (LMICs) is striking with an estimated 5 billion people lacking access to surgical care when needed[1,2] and the burden among children similarly high[2–4]. Many pediatric conditions requiring surgery carry the risk of life-long disability or higher risk of mortality given that the conditions arise during the years of critical development[5]. Thus, children represent a population with unique surgical needs, including anesthetic, perioperative, and postoperative capacity requirements.

While recent attention to global surgery has been expanding, the burden of conditions requiring surgery among the highly vulnerable pediatric age group is relatively unknown, particularly within LMICs. Although children make up approximately 50% of the total population in LMICs, there is little focus on childhood surgical conditions and attention has instead been on infectious diseases and obstetric care[6]. Although these efforts aimed on reducing infectious diseases and increasing obstetric care are certainly notable, approximately 85% of children in LMICs will have a surgically-treatable condition by the age of 15[2], thus highlighting the immense need for research and interventions aimed at reducing these burdens and providing quality surgical care when needed.

In the recent landmark publication, Lancet Global Surgery 2030[1], researchers outlined a goal of providing safe, affordable surgical and anesthetic care in LMICs. Precise and accurate identification of the burden of pediatric surgical need is essential to attain this goal and can assist healthcare workers and policy makers in identifying areas with the most need. Likewise, quantification of the pediatric burden will inform targeted and tailored interventions in LMICs. Previous attempts at quantifying pediatric surgical burden have been done within healthcare systems[7], which neglect to include those without access to the healthcare system. Only 6.3% of surgical procedures conducted worldwide occurred in LMICs. Thus, hospital-based estimates of children in need of surgical care likely underestimates the needs for the population as a whole since many children never reach the hospital for care. A community-based estimate would provide a more refined and accurate estimate of the global pediatric surgical burden.

The Surgeons OverSeas Assessment of Surgical Need (SOSAS) tool was developed to provide a population-based household survey assessing the prevalence of surgically treatable conditions in LMICs and has been conducted in Nepal, Sierra Leone, Rwanda, and Uganda. We evaluated the surveys within these countries to quantify the pediatric surgical need and identify predictors of treated surgical conditions.

## Materials and methods

Data was collected by the SOSAS survey, a validated cross-sectional, cluster-based population survey in four countries: Nepal, Rwanda, Sierra Leone, and Uganda[8–11]. The survey was administered in Rwanda in 2011, Sierra Leone in 2012, Nepal in 2014, and Uganda in 2014. Nepal, Rwanda, Sierra Leone, and Uganda are low-income countries with vastly different child health indicators than the United States (Table 1). Life expectancy is lower than the United States by 29 years in Sierra Leone, 19 years in Uganda, 16 years in Rwanda, and 10 years in Nepal. The percentage of the population under the poverty line is much higher in all 4 countries, with the highest percentage in Sierra Leone (53%) followed by Rwanda (23%). Neonatal, infant, and under-5 mortality rates per 1,000 live births are 5 to 17 times higher than the US rates. Compared to the US population where 19% is under the age of 15, 34% to 48% of the 4 LMIC populations are under 15.

**Table 1:** Child health indicators and surgery providers in 4 low- and middle-income countries compared to US.

	Nepal	Rwanda	Sierra Leone	Uganda	United States
Life expectancy at birth (years)^12^	69	63	50	58	79
Poverty headcount ratio at national poverty lines (%)^12^	25.2	44.9	52.9	19.5	14.5
Neonatal mortality rate (per 1000 live births)^12^	22	19	35	19	4
Infant mortality rate (per 1000 live births)^12^	29	31	87	38	6
Under-5 mortality rate (per 1000 live births)^12^	36	42	120	55	7
Population under 15 years of age (%)^12^	34	41	43	48	19
Surgical physician density (per 100,000 population)^15^	NA	0.49	0.18	0.73	38
Pediatric surgeons (number)*	NA	1	1	3	1,150[16]

NA: data not available.

*Personal communications with local surgical partners.

Modifications to the SOSAS survey within each country were minimal with the exception of language adaptations and the addition of a physical examination in the Nepalese survey for validation of the survey[11]. Survey clusters were randomly selected in a two-stage process with a probability adjustment for population size and geographic stratification into rural and urban populations. Physicians, medical interns, and students from medical, nursing, and public health schools (Rwanda, Nepal, and Sierra Leone) or experienced data collectors (Uganda) were trained to administer the survey to separate households within the 4 countries. During the study period, collected data was reviewed by field supervisors to ensure quality of data and identify any data collection inconsistencies between interviewers. The survey contained components capturing household level characteristics, such as household demographics and information on deaths within the household during the previous 12 months, individual level characteristics, such as the presence of surgical conditions, clinical characteristics of surgical conditions, healthcare seeking behavior, and area level characteristics, such as availability of treatment facilities.

Children were defined as persons between the ages of 0 to 18 years. The head of household and each individual participant prior to initiating the survey provided informed consent. A parent or guardian provided consent for all participants younger than 18 years old, and children ages 8 to 18 provided assent. For the majority of children, parents answered questions about their children. For older children, who were asked to answer sensitive reproductive health questions, parents assisted in answering questions if the child assented to having a parent present for the interview.

A surgical need was self-reported (with the assistance of parents when necessary) by the survey respondents and included all cases that were considered to need a surgical consultation. Healthcare facilities were defined as primary (a facility without a functioning operating room), secondary (a facility with a functioning operating room), and tertiary (a facility with a functioning operating room and a minimum of a surgical specialist, such as a general surgeon, orthopedic surgeon, gynecologist, or urologist). Care provided at a healthcare facility by a physician or a nurse was defined as modern healthcare, while care provided by a traditional healer outside of a healthcare facility was defined as traditional health care. A minor surgical procedure was defined as a procedure that required no anesthesia or local anesthesia and major surgical procedure was defined as a procedure requiring regional or general anesthesia. Met need covered any surgically-treatable condition that was reported as having had surgical care, minor or major, in the past. Unmet need refers to any condition that is surgically treatable, and the respondent did not seek healthcare, sought healthcare but could not receive surgical care, or had persistent, untreated complications/sequela from receiving surgical care. Transportation costs in Rwandan francs, Sierra Leonean leones, Nepalese rupees, and Ugandan shillings were converted to US dollars and set to equivalent 2016 value.

Data analysis was performed using STATA (StataCorp, College Station, TX, USA). Demographic data, clinical characteristics of surgical needs, and healthcare seeking behavior variables were compared between the 4 countries, using a weighted model to account of sampling biases and cluster sampling design within each country. Significance testing was achieved by Χ^2^ or ANOVA and set at p<0.05. Predictors of having a met surgical condition were analyzed through a multivariate logistic regression model with reported odds ratios and 95% confidence intervals. Research ethics approval was obtained from the scientific review boards of each country, as well as the collaborating institutions for each country. (Nepal Medical College, Kathmandu, Nepal; University of California-San Francisco, East Bay, Oakland, California; University of Rwanda, Kigali, Rwanda; University of Virginia, Charlottesville, Virginia; Johns Hopkins Bloomberg School of Public Health, Baltimore, Maryland; Columbia University, New York, New York, Stanford University, Stanford, California; Connaught Hospital, Freetown, Sierra Leone, College of Medicine and Allied Health Science, Freetown, Sierra Leone; Johns Hopkins Hospital, Baltimore, Maryland; Uganda National Council for Science and Technology, Kampala, Uganda; Makerere University School of Medicine, Kampala, Uganda; Duke University Health System, Durham, North Carolina; and University of Minnesota, Minneapolis, Minnesota.)

## Results

A total of 13,806 total respondents were surveyed in Nepal (96.8% of eligible household responded), Rwanda (99.9% of eligible households responded), Sierra Leone (98.3% of eligible household responded), and Uganda (96.4% of eligible households responded). Of these SOSAS survey respondents, 6,361 (46.1%) were children (Table 2). Children in Rwanda were slightly younger than the other countries with a median age of 7 years (interquartile range [IQR] 3–11) and children in Nepal were slightly older with a median age of 10 years (IQR 5–15) (p-value <0.0001). There were similar proportions of males to females in each country with 51.5% male and 48.5% female, overall (p-value 0.9). Over 75% of all survey respondents reported living in rural areas with the greatest percentages in Rwanda (90.6%) and Uganda (81.8%) followed by Nepal (71.0%) and Sierra Leone (62.9%) (p-value <0.0001). The median number of persons within each household ranged from 5 members in Nepal, Rwanda, and Uganda to 6 members in Sierra Leone (p-value <0.0001).

**Table 2:** Demographic and healthcare facility characteristics and healthcare seeking behaviors among pediatric SOSAS survey respondents, by country.

	TOTAL	Nepal	Rwanda	Sierra Leone	Uganda	*P* value*
**Demographic characteristics**						
Number of children (% of total respondents)	6,361 (46.1)	799 (39.7)	1,683 (52.9)	1,703 (46.2)	2,176 (51.2)	<0.001
Age of children, median years (IQR)	8 (4–13)	10 (5–15)	7 (3–11)	8 (4–14)	8 (4–13)	<0.001
Age categories of children, number (%)						
0–5 years	2,294 (36.0)	230 (28.8)	696 (41.3)	560 (32.9)	808 (37.1)	<0.001
6–9 years	1,399 (22.0)	143 (17.9)	403 (24.0)	381 (22.4)	472 (21.7)	
10–14 years	1,492 (23.5)	204 (25.5)	365 (21.7)	392 (23.0)	531 (24.4)	
15–18 years	1,176 (18.5)	222 (27.8)	219 (13.0)	370 (21.7)	365 (16.7)	
Sex of children, number (%)						
Male	3,277 (51.5)	478 (59.8)	869 (51.6)	847 (49.7)	1,083 (49.8)	0.917
Female	3,084 (48.5)	321 (40.2)	814 (48.4)	856 (50.3)	1,093 (50.2)	
Village type, number (%)						
Rural	4,944 (77.7)	567 (71.0)	1,525 (90.6)	1,072 (62.9)	1,780 (81.8)	<0.001
Urban	1,417 (22.3)	232 (29.0)	158 (9.4)	631 (37.1)	396 (18.2)	
Number in household, median, (range)	5 (4–7)	5 (2–15)	5 (1–12)	6 (2–24)	5 (1–19)	<0.001
Surgical need, number (%)						
No	5,180 (81.5)	658 (82.4)	1,484 (88.2)	1,235 (72.5)	1,803 (82.9)	<0.001
Yes	1,181 (18.5)	141 (17.6)	199 (11.8)	468 (27.5)	373 (17.1)	
Met or unmet surgical need, number (%)**						
Met need	443 (37.5)	82 (58.2)	91 (45.7)	139 (29.7)	131 (35.1)	<0.001
Unmet need	738 (62.5)	59 (41.8)	108 (54.3)	329 (70.3)	242 (64.9)	
**Primary healthcare facility characteristics**						
Main transport mechanism, number (%)						
Motorized	1,473 (23.2)	72 (9.0)	204 (12.1)	345 (20.3)	852 (39.2)	<0.001
Non-motorized	4,888 (76.8)	727 (91.0)	1,479 (87.9)	1,358 (79.7)	1,324 (60.8)	
Availability of transport funds, number (%)						
Yes	3,240 (50.9)	90 (11.3)	1,186 (70.5)	249 (14.6)	1,715 (78.8)	<0.001
No or No Replies/Don’t Know	3,121 (49.1)	709 (88.7)	497 (29.5)	1,454 (85.4)	461 (21.2)	
**Healthcare seeking behaviors,** number (%)						
Reasons for not seeking care when needed***						
No money for healthcare or transport	410 (61.9)	15 (23.8)	33 (30.3)	315 (77.8)	47 (55.3)	<0.001
No facility available	112 (16.9)	22 (34.9)	66 (60.6)	24 (5.9)	0 (0.0)	

*Χ^2^ for dichotomous variables and ANOVA for 3 or more levels.

**Among those reporting a surgical need. A child was deemed as having unmet need if at least one of their surgical needs was unmet.

***Among those with unmet need and provided a reason (N = 662).

Overall, 18.5% of children had a surgical need and 62.5% of these children had at least one unmet need. The highest need for pediatric surgical care was in Sierra Leone with 27.5% of children reporting at least one surgical condition, followed by Nepal (17.6%), Uganda (17.1%), and Rwanda (11.8%). Among children with surgical conditions, 70.3% in Sierra Leone had at least one unmet need, followed by 64.9% in Uganda, 54.3% in Rwanda, and 41.8% in Nepal.

In all four countries, the most common method of reaching a primary healthcare facility was through non-motorized transportation such as walking, being carried, or by animal. The availability of funds necessary for transportation was highest in Rwanda and Uganda (70.5% and 78.8%, respectively) and much lower in Nepal and Sierra Leone (11.3% and 14.6%, respectively) (p-value <0.0001).

**Fig 1:**
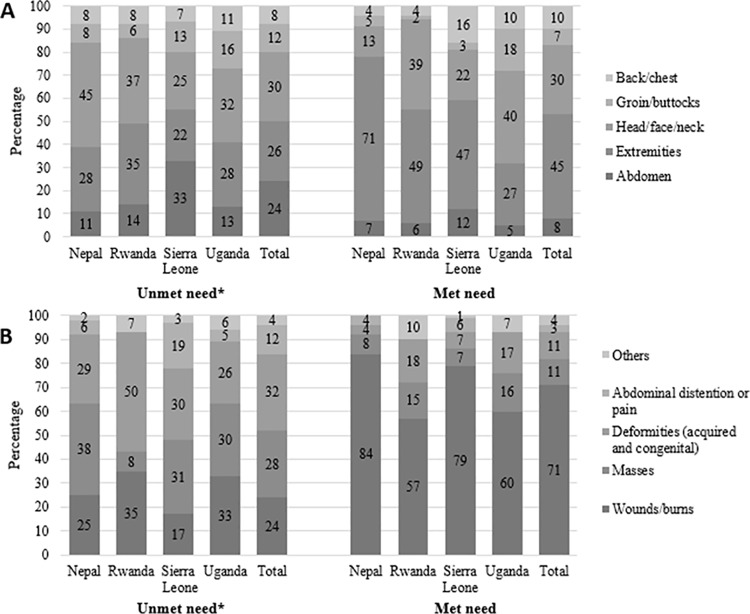
**(A) Anatomic region and (B) type of surgical conditions in Nepal, Rwanda, Sierra Leone, and Uganda.** *Unmet need: There were 755 conditions among 738 children. Met need: There were 575 conditions among 443 children. NOTE: Met need was defined as any surgically-treatable condition that was reported as having had surgical care, minor or major, in the past. Unmet need was defined as any condition that is surgically treatable, and the respondent did not seek healthcare, sought healthcare but could not receive surgical care, or had persistent, untreated complications/sequela from receiving surgical care. Percentages are based on number of conditions in each category divided by the total number of unmet/met conditions in the respective country.

Fig 1 breaks down surgical conditions by anatomic region and type of condition, stratified by country and unmet versus met need (Fig 1). In all countries except Sierra Leone, the highest percentage of unmet surgical conditions was head, face, and neck conditions, followed by conditions in the extremities. In Sierra Leone, abdominal conditions were most common, followed by head, face, and neck conditions. Nearly half of met surgical conditions were in the extremities. In Nepal and Rwanda, 71% and 49% of all conditions that were treated occurred in the extremities, followed by face, head, and neck conditions (13% and 39%, respectively). Extremity conditions were more likely to be treated and abdomen conditions were more likely to be untreated in all countries, with the largest differential in Sierra Leone (33% unmet versus 12% met need).

In three of the four countries, over a third of the untreated conditions were masses. In Rwanda, only 8% of untreated conditions were attributed to masses. In Nepal, 38% of the untreated surgical conditions were masses, 29% were acquired or congenital deformities, and 25% were wounds or burns, while 84% of the treated conditions were for wounds or burns. In Rwanda, 50% of untreated conditions were attributed to deformities, and 35% were wounds or burns. In Sierra Leone and Uganda, one-third of untreated conditions were attributable to masses or deformities, and 17% (Sierra Leone) or 33% (Uganda) were attributable to wounds or burns. The overwhelming majority of treated conditions in all countries were for wounds or burns.

## Discussion

This study combines data from four low-income countries to better elucidate the burden of surgical disease in pediatric populations in these LMICs. Overall, there is a high burden of unmet surgical need in the pediatric population of these countries. Nearly 20% of children had a surgical need, and 62% of those children had at least one unmet surgical need. Extrapolating these estimates to the current pediatric populations in the four countries equates to an estimated 3.7 million children in need of surgical care[12].

While historically surgery has been deemed too resource intensive to merit significant investment, selected pediatric surgical interventions such as cleft lip or palate repair ($48/disability-adjusted life year [DALY] averted), general surgery ($82/DALY averted), or hydrocephalus repair ($108/DALY averted) are similarly cost-effective compared to antiretroviral therapy for HIV ($550/DALY averted) or BCG vaccine for prevention of tuberculosis ($120/DALY averted)[1,13]. The global health community as well as local governments must make investments in improving access to surgical care to close the gap of unmet surgical need among children in LMICs.

Existing data indicates severe shortages in surgical, anesthetic, and obstetric providers in LMICs[1] with over 1 million positions unfilled. Thus, current provision of pediatric surgical care in these low-income countries is generally limited to general medical officers and general surgeons. These surgeons practice predominantly within large tertiary care facilities, which a large portion of the population is unable to reach[14]. In this study, wounds, burns, and extremity conditions were more likely to be treated compared to congenital deformities, abdominal conditions, or head and neck conditions. General practitioners can meet much of the pediatric surgical need including burns, wound debridement, and suturing. However, conditions requiring more specialized care, such as congenital deformities, intra-abdominal tumors, and hydrocephalus require a specialized pediatric surgeon.

There is a large deficiency in providers trained in pediatric surgical care in areas of great need, with many countries lacking any providers[7]. In our study, unmet pediatric surgical needs were highest in Sierra Leone, a country with no pediatric surgeons and a surgical physician density of 0.18 per 100,000 people[15]. Similarly, 17% of children in Uganda had a surgical need but reside in a country with 4 practicing pediatric surgeons and a surgical physician density of 0.73 surgeon physicians per 100,000 people. Similar conclusions can be drawn for Nepal and Rwanda, countries with fewer than 1 surgical physician or pediatric surgeon per 100,000 people. In addition to provider shortages, there is also a maldistribution of the surgical workforce with most of the doctors concentrated in urban areas[15]. This contributes to a lack of access to surgical care for children due to lengthy travel and poor road conditions. In our study, 78% of children lived in rural areas, highlighting the great need to increase access in remote areas throughout LMICs.

This study has several limitations. As previously discussed[8–11], the SOSAS survey is a household based survey that relies on self-reported symptoms and patient recollection of medical care. There is a lack of specificity to surgical disease descriptions, which does not allow for specifically identifying types of surgical disease. The SOSAS survey is able to identify need for surgical consultation but cannot provide accurate and specific enough data to clearly identify how many pediatric surgeons would be adequate to meet the need in low-income countries. Although reliance on self-reporting is a criticism of population-based health assessment methodologies, the SOSAS assessment was compared with a visual examination by a physician with agreement in 94.6% of cases[11].

The pediatric population is growing in low-income countries where over 50% of the population is less than 15 years of age. As such, progress in delivery of surgical care will require specifically targeting delivery of pediatric surgical care. This analysis of the SOSAS survey in four low-income countries is a starting point to identify that there is a large unmet need and investments must be made to increase surgical capacity, decrease barriers to care, and develop innovative solutions to provide pediatric surgical care in these resource limited settings.

## Supporting information

S1 File Survey_English_SOSAS 2014 04 20. (DOCX)

## References

[1] Meara JG, Leather AJ, Hagander L, Alkire BC, Alonso N, Ameh EA, et al Global Surgery 2030: evidence and solutions for achieving health, welfare, and economic development. Lancet (London, England). 2015;386(9993):569–624. 10.1016/S0140-6736(15)60160-X 25924834

[2] Bickler SW, Rode H. Surgical services for children in developing countries. Bulletin of the World Health Organization. 2002;80(10):829–35. 12471405 PMC2567648

[3] Butler EK, Tran TM, Fuller AT, Brammell A, Vissoci JR, de Andrade L, et al Quantifying the pediatric surgical need in Uganda: results of a nationwide cross-sectional, household survey. Pediatric surgery international. 2016. 10.1007/s00383-016-3957-3 PMC5050237 27614904

[4] Fuller AT, Haglund MM, Lim S, Mukasa J, Muhumuza M, Kiryabwire J, et al Pediatric Neurosurgical Outcomes Following a Neurosurgery Health System Intervention at Mulago Hospital in Uganda. World neurosurgery. 2016. 10.1016/j.wneu.2016.07.090 27497624

[5] Ozgediz D, Poenaru D. The burden of pediatric surgical conditions in low and middle income countries: a call to action. Journal of pediatric surgery. 2012;47(12):2305–11. 10.1016/j.jpedsurg.2012.09.030 23217895

[6] Nations U. The Millennium Development Goals Report 2015. New York; 2015.

[7] Greenberg SL, Ng-Kamstra JS, Ameh EA, Ozgediz DE, Poenaru D, Bickler SW. An investment in knowledge: Research in global pediatric surgery for the 21st century. Seminars in pediatric surgery. 2016;25(1):51–60. 10.1053/j.sempedsurg.2015.09.009 26831138

[8] Groen RS, Samai M, Stewart KA, Cassidy LD, Kamara TB, Yambasu SE, et al Untreated surgical conditions in Sierra Leone: a cluster randomised, cross-sectional, countrywide survey. Lancet (London, England). 2012;380(9847):1082–7. 10.1016/S0140-6736(12)61081-2 22898076

[9] Petroze RT, Calland JF, Niyonkuru F, Groen RS, Kyamanywa P, Li Y, et al Estimating pediatric surgical need in developing countries: a household survey in Rwanda. Journal of pediatric surgery. 2014;49(7):1092–8. 10.1016/j.jpedsurg.2014.01.059 24952795

[10] Fuller AT, Butler EK, Tran TM, Makumbi F, Luboga S, Muhumza C, et al Surgeons OverSeas Assessment of Surgical Need (SOSAS) Uganda: Update for Household Survey. World J Surg. 2015. 10.1007/s00268-015-3191-5 26316109

[11] Gupta S, Shrestha S, Ranjit A, Nagarajan N, Groen RS, Kushner AL, et al Conditions, preventable deaths, procedures and validation of a countrywide survey of surgical care in Nepal. The British journal of surgery. 2015;102(6):700–7. 10.1002/bjs.9807 25809125

[12] The World Bank Data Catalog: World Bank; [Available from: http://datacatalog.worldbank.org/.

[13] Saxton AT, Poenaru D, Ozgediz D, Ameh EA, Farmer D, Smith ER, et al Economic Analysis of Children's Surgical Care in Low- and Middle-Income Countries: A Systematic Review and Analysis. PloS one. 2016;11(10):e0165480 10.1371/journal.pone.0165480 27792792 PMC5085034

[14] O'Flynn E, Andrew J, Hutch A, Kelly C, Jani P, Kakande I, et al The Specialist Surgeon Workforce in East, Central and Southern Africa: A Situation Analysis. World journal of surgery. 2016. 10.1007/s00268-016-3601-3 27283189

[15] Hoyler M, Finlayson SR, McClain CD, Meara JG, Hagander L. Shortage of doctors, shortage of data: a review of the global surgery, obstetrics, and anesthesia workforce literature. World journal of surgery. 2014;38(2):269–80. 10.1007/s00268-013-2324-y 24218153

[16] Nakayama DK, Burd RS, Newman KD. Pediatric surgery workforce: supply and demand. Journal of pediatric surgery. 2009;44(9):1677–82. 10.1016/j.jpedsurg.2009.03.036 19735808

