# Supplementary material for: Epidemiology of pediatric surgical needs in low-income countries
Source: PLoS One. 2017 Mar 3;12(3):e0170968. doi: 10.1371/journal.pone.0170968 (PMC5336197; doi:10.1371/journal.pone.0170968)
Supplement: S1 File — (DOCX) [file pone.0170968.s001.docx]

**Surgeons OverSeas Assessment of Surgical Need (SOSAS) Version 3.0**

**Items in bold are the new questions.**

Under the bold items the questions to be asked.

*[Items in italics are instructions to the interviewer.]*

| Paragraphs in the boxes are explanations during the interview for the respondent. Read these out loud and be sure that the person understands the explanation. |
| --- |

A. HOUSEHOLD INFORMATION

*[A1-A5 to be completed by the interviewer]*


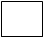
**A1. Number of visits:** _____ 1 _____ 2 _____ 3 _____ 4

*[Check off each visit to the household, check the box at the end if this is a replaced household]*

**A2. Village # OR Cluster #:** _____________________

**A3. Village Type:** ____ Rural _____ Urban _____ Slum

**A4. Household Code:** ______________

**A5. Interviewer Name:** ______________

*[The self-designated head of household will answer sections A-D. First identify the head of household and then proceed.]*

| Good morning/evening. My name is [*Give your name*]. I am a member of the village health team and we are working with Makerere University and the Ministry of Health [*show the information letter*].  We are wondering if you would like to participate in a research study to find out if there are enough doctors in the area, particularly if there are enough surgeons. A surgeon is a medical doctor who cures patients by taking care of wounds and broken bones or cutting out masses. Sometimes surgeons must put you to sleep to do these things, and other times they must only numb the hurt body part.  You were selected as a possible participant because you live in one of the districts we want to learn more about. I want to take a couple minutes to tell you more about this study. Please ask me questions at any time if you do not understand something that I say.  Dr. Moses Galukande, a surgeon from Makerere University, is working with 3 other surgeons: Dr. Samuel Luboga from Makerere University, Dr. Michael Haglund from Duke University in the USA, and Dr. Jeffrey Chipman from the University of Minnesota in the USA. Dr. Mukumbi Fred from Makerere University, who is a specialist in doing studies to learn about an entire population of people, is also working on this study. The study is funded by [*will insert funding once confirmed.].*  We are doing this study to find out what kinds of medical problems are common in Uganda, particularly problems that could be treated by surgery. We want to find out what kinds of things make it difficult for people living in your area to get health care when needed.  To find out if there are enough doctors taking care of these problems in your area, we'd like to ask you and two other members of your household some questions. If you agree to participate in this study, we would ask you to answer some questions about you and hour household. This survey will take about 30 minutes to 1 hour. First, I will ask you some questions about the people who live in this house and any people who used to live in this house who have died in the last year. After that, I will randomly choose two people from your household and ask them more detailed questions about their health. We won’t be offering any medical care right now, but we hope that the information you provide will help bring improved services in the future.  The study has the following risks. We want to make sure that you understand that we are not offering medical care right now. We are not doctors and are not experts in surgery. If we uncover a health problem that you or one of your family members has that might need medical care, we will refer you or your family member to the nearest health facility that has the capabilities that you need. I will also ask you to tell me detailed information about your health. You may feel uncomfortable answering some of these questions. I will ask you the questions in a private area where no one will hear your responses except me. I will document your responses on this tablet, but I will never record your name or address. No one will know how you answered any of the questions. If at any time you are too uncomfortable to answer one of the questions, you are welcome to not answer that question or stop the survey altogether. I will also ask you to recall information about members of your household who have died in the last year. We understand that it can be difficult to talk about people who have died recently. If at any time you are too uncomfortable to talk about this, you are welcome to not answer that question or stop the survey altogether.  There are a couple possible direct benefits to you by participating in this study. First, if we do uncover that you have a problem that could be treated by surgery, we can refer you to the closest health facility that can take care of you. Second, by participating in this survey you will learn some information about problems that could possibly be treated by surgery. With this knowledge, you may be able to better recognize if you or one of your family members needs to go to a health center. The main benefit of participating in this survey is that the information you provide can help the Ministry of Health bring more skilled doctors to your area.  The information you provide will be kept private. No information about your identity including your name, address, or birthday will be recorded. The researchers conducting this study and the Uganda Ministry of Health will have access to the information you provide but again, they will not know that it was you who provided the information. In any publications or presentations, the researchers will not include any information that will make it possible to identify you as a study participant. The information you provide will be sent over the internet to the researchers conducting this study, but it will be kept safe with passwords and protections to keep other people from looking at it.  You do not have to participate in the study if you do not want to. If you decide to participate, you are free to stop at any time.  If you have any questions, you may ask them now. If you have questions later, you are encouraged to contact the researchers with the contact information given on this information sheet. This sheet has all of the information we just talked about *[Give the head of household the information sheet]*. Do you have any questions at the moment? *[Answer any questions they have. If there are questions you cannot answer, contact your Field Supervisor before proceeding.]*  *[Test the subject’s comprehension of the study.]* Now in order to make sure you understand the study, I want to ask you a few simple questions. What are we asking you to do by participating in this study? *[Pause to allow them to answer. Do not go on until you have an adequate answer.]* Why are we asking you questions about your health? *[Pause to allow them to answer. Do not go on until you have an adequate answer.]* |
| --- |

**A6. Informed consent:**

Do you understand what we have talked about and have you had all of your questions answered? Do you consent to participation in this study?

___ Yes

___ No (If no, what is the reason? _____________)

(No time / no willingness / no reason / no seen benefit / other: explain….)

*[Without informed consent you cannot proceed. Make sure the person understands the purpose of this survey. If they don’t want to participate, ask why and mark this.]*

B. LIVING HOUSEHOLD MEMBERS

| The following questions will be about your household members. I want to include every person that you consider to be part of your household. We will start the information of the oldest household member and finish with the youngest, also babies, newborns, and disabled household members need to be listed in order of their age. |
| --- |

*[Fill in all the household members’ age and sex in the table, ordered by age, the oldest household person first. Also the household members who are disabled or ill should be mentioned. At the end of the list of household members you need to ask specifically for the newborns and babies in the family and collect the information for each of them in individual tabs.]*

**B0. Number of household members:**

In total, how many people live in your household? _____

*[*Age for babies:*

*<3 months = 0*

*3 - < 6 months = 0.25*

*6 - < 9 months = 0.5*

*9 - < 12 months = 0.75*

*12 - < 24 months = 1 etc.]*

| **ID Number** | **B1. Age:**  (years*) | **B2. Sex:**  (Male = M/ Female = F) |
| --- | --- | --- |
| 1 |  |  |
| 2 |  |  |
| 3 |  |  |
| 4 |  |  |
| 5 |  |  |
| 6 |  |  |
| 7 |  |  |
| 8 |  |  |
| 9 |  |  |
| 10 |  |  |
| 11 |  |  |
| 12 |  |  |
| 13 |  |  |
| 14 |  |  |
| 15 |  |  |
| 16 |  |  |
| 17 |  |  |
| 18 |  |  |
| 19 |  |  |
| 20 |  |  |
| 21 |  |  |
| 22 |  |  |
| 23 |  |  |
| 24 |  |  |
| 25 |  |  |
| 26 |  |  |
| 27 |  |  |
| 28 |  |  |
| 29 |  |  |
| 30 |  |  |

*Step 1. Check if all information is filled in for all the household members.*

*Step 2. The total number of household members should be the same as the total number ages and sexes you filled in for question B.*

*Step 3. Check the order of the household members. The list should start with the oldest person and end with the youngest person. Make sure you ask specifically for neonates /babies.*

*Step 4. Remember the total number of living household members. Open your Random Generator on your iPad: fill in for 'Min':1 and for 'Max' : the number of living household members. Press Generate. The first number which appears corresponds with the number of the first household member to interview. Repeat the procedure also to assign the person for the second interview.*

ID number of the assigned household member for the first interview: _____

ID number of the assigned household member for the second interview: _____

C. TRANSPORTATION MEANS

| The following questions will be about the health facilities available to you and your household members and the transportation you are able to provide for them in need of health care. |
| --- |

**C1.1. TRANSPORT TO PRIMARY HEALTH FACILITY (typically HCII or HCIII):**

What is the main way for you or your household members to go to the closest Health Center II or Health Center III?

___ Public transport: bus

___ Public transport: taxi

___ Public transport: boda boda

___ Private Car

___ Private Motorcycle

___ Bicycle

___ Boat

___ Animal

___ On foot

___ Carried

*[Use the name of the village/city where this facility is found for the person to be able to respond to questions C1.2 to C1.4;*

*Primary health facility is defined as a health facility without functioning operating theatre;*

*Time guideline: one person can walk 3 miles in one hour or 1 mile takes 20 minutes to walk]*

**C1.2. Travel time to primary health facility:**

How long does it take you in total to get to your primary health facility if you don't have to wait for transportation? (minutes)

_____

**C1.3. Waiting time for transport:**

How long do you probably have to wait for transportation to a primary health facility? (minutes)

_____

**C1.4. Cost for transport:**

What does it cost you to provide transportation to a primary health facility for a sick household member? (UGX)

_____ 0 – 500

_____ 501 – 1,000

_____ 1,001 – 2,000

_____ 2,001 – 5,000

_____ 5,001 – 10,000

_____ 10,001 – 20,000

_____ 20,001 – 50,000

_____ 50,001 – 100,000

_____ >100,000

**C1.5. Transport money available?**

Are you always able to provide these means for transport of a sick household member?

_____ Yes

_____ No

_____ N/A

**C2.1. TRANSPORT TO SECONDARY HEALTH FACILITY (typically HCIV or District Hospital):**

What is the main way for you or your household members to go to the closest Health Center IV or District Hospital?

___ Public transport: bus

___ Public transport: taxi

___ Public transport: boda boda

___ Private Car

___ Private Motorcycle

___ Bicycle

___ Boat

___ Animal

___ On foot

___ Carried

*[Use the name of the village/city where this facility is found for the person to be able to respond to questions C2.2 to C2.4;*

*Secondary health facility: Health facility with functioning operating theatre; if the closest HCIV does not have a functioning operating theatre, then ask about the closest District Hospital;*

*Time guideline: one person can walk 3 miles in one hour or 1 mile takes 20 minutes to walk]*

**C2.2. Travel time to secondary health facility:**

How long does it take you in total to get to your secondary health facility if you don't have to wait for transportation? (minutes)

_____

**C2.3. Waiting time for transport:**

How long do you probably have to wait for transportation to a secondary health facility? (minutes)

_____

**C2.4. Cost for transport:**

What does it cost you to provide transportation to a secondary health facility for a sick household member? (UGX)

_____ 0 – 500

_____ 501 – 1,000

_____ 1,001 – 2,000

_____ 2,001 – 5,000

_____ 5,001 – 10,000

_____ 10,001 – 20,000

_____ 20,001 – 50,000

_____ 50,001 – 100,000

_____ >100,000

**C2.5. Transport money available?**

Are you always able to provide these means for transport of a sick household member?

_____ Yes

_____ No

_____ N/A

**C3.1. TRANSPORT TO TERTIARY HEALTH FACILITY (typically Regional or National Referral Hospitals):**

What is the main way for you or your household members to go to the closest Regional or National Referral Hospital?

___ Public transport: bus

___ Public transport: taxi

___ Public transport: boda boda

___ Private Car

___ Private Motorcycle

___ Bicycle

___ Boat

___ Animal

___ On foot

___ Carried

*[Use the name of the village/city where this facility is found for the person to be able to respond to questions C3.2 to C3.4;*

*Tertiary health facility: Health facility with functioning operating room and at least one surgical specialis (Surgeons/Orthopedics/Gynecologist/Urologist);*

*Time guideline: one person can walk 3 miles in one hour or 1 mile takes 20 minutes to walk]*

**C3.2. Travel time to tertiary health facility:**

How long does it take you in total to get to your tertiary health facility if you don't have to wait for transportation? (minutes)

_____

**C3.3. Waiting time for transport:**

How long do you probably have to wait for transportation to a tertiary health facility? (minutes)

_____

**C3.4. Cost for transport:**

What does it cost you to provide transportation to a tertiary health facility for a sick household member? (UGX)

_____ 0 – 500

_____ 501 – 1,000

_____ 1,001 – 2,000

_____ 2,001 – 5,000

_____ 5,001 – 10,000

_____ 10,001 – 20,000

_____ 20,001 – 50,000

_____ 50,001 – 100,000

_____ >100,000

**C3.5. Transport money available?**

Are you always able to provide these means for transport of a sick household member?

_____ Yes

_____ No

_____ N/A

D. DECEASED HOUSEHOLD MEMBERS

**D0. Number of household deaths:**

Did you have any household member who passed away in the past year? I'm very sorry to hear that, how many people from your household died in the past year? ______

[*Ask specifically for neonates and babies. If yes, continue in this section; if no, skip to the next section.*]

| ‘I'm sorry to hear that you lost a household member in the last year. The following questions are about this/these deceased person(s).’ |
| --- |

| 1….. | 2….. | 3….. | 4….. | 5….. |
| --- | --- | --- | --- | --- |
|  |  |  |  |  |

**D1. Age at death:**

How old was the household member when she/he died?

*[Age for babies:*

*<3 months = 0*

*3 - <6 months = 0.25*

*6 - <9 months = 0.5*

*9 - <12 months = 0.75*

*12 -<24 months = 1 etc.]*

| 1….. | 2….. | 3….. | 4….. | 5….. |
| --- | --- | --- | --- | --- |
|  |  |  |  |  |
|  |  |  |  |  |

**D2. Sex:**

What was the sex of the household member? Male

Female

**D2.1 Pregnant at death:** Yes = 1 No = 2 *[Answer only if D2 answer is Female]*

| 1….. | 2….. | 3….. | 4….. | 5….. |
| --- | --- | --- | --- | --- |
|  |  |  |  |  |

Was the household member pregnant when she died or did she deliver within 6 weeks of her death?

**D3. Death specifics:**

Did the household member have any of the following problems less than a week before s/he died?

For neonates / babies: Did the child look normal and could it drink, urinate and defecate normally after birth? *[If no, ask further questions to determine if there was a congenital deformity]*

| 1….. | 2….. | 3….. | 4….. | 5….. |
| --- | --- | --- | --- | --- |
|  |  |  |  |  |
|  |  |  |  |  |
|  |  |  |  |  |
|  |  |  |  |  |
|  |  |  |  |  |
|  |  |  |  |  |
|  |  |  |  |  |
|  |  |  |  |  |

*[Show empathy for the story told. Don't be judgmental in any way; let the person tell his/her story. For definitions see Question F2.1]*

Wound or infection due to injury

Wound or infection not due to an injury

Bleeding or ill around childbirth

Mass (Growth or Swelling)

Deformity congenital

Deformity acquired

Abdominal distention or pain

None of the above

If None of the above, write in cause of death for each number of deaths

1. ________________________________________________________________________________________
2. ________________________________________________________________________________________
3. ________________________________________________________________________________________
4. ________________________________________________________________________________________
5. ________________________________________________________________________________________

**D3.1 Type of injury / accident:**

Did the problem start after an injury or accident? What kind of accident?

| 1….. | 2….. | 3….. | 4….. | 5….. |
| --- | --- | --- | --- | --- |
|  |  |  |  |  |
|  |  |  |  |  |
|  |  |  |  |  |
|  |  |  |  |  |
|  |  |  |  |  |
|  |  |  |  |  |
|  |  |  |  |  |
|  |  |  |  |  |
|  |  |  |  |  |
|  |  |  |  |  |

*[Pick the one that best describes the injury / accident. Pedestrian and bicycle crash definition: there was no motorized vehicle involved. All can be intentional or unintentional.]*

No, it was not due to an injury / accident

Car, truck, bus crash

Motorcycle crash

Pedestrian, bicycle crash

Gunshot / firearm

Stab / slash / cut / crush

Bite or animal attack

Fall

Open fire / explosion

Hot liquid / hot object

| 1….. | 2….. | 3….. | 4….. | 5….. |
| --- | --- | --- | --- | --- |
|  |  |  |  |  |
|  |  |  |  |  |

**D4.1 Healthcare sought:**

Did the household member go to a health facility or see a doctor/nurse before she/he died? Yes

No

**D4.2 Type of healthcare received:** *[only if D4.1 is yes]*

What kind of treatment did the household member receive? *[A major procedure is a procedure which requires regional/spinal/general anesthesia; A minor procedures is a procedure requiring local anaesthesia, dressings, wound care, punctures, suturing or incision and drainage]*

| 1….. | 2….. | 3….. | 4….. | 5….. |
| --- | --- | --- | --- | --- |
|  |  |  |  |  |
|  |  |  |  |  |
|  |  |  |  |  |

None / No surgical care

Major procedure

Minor procedures

**D4.3 Reason for not having surgical care:** *[Only if D4.1 is ‘No’ or D4.2 is ‘No surgical care’]*

What was the main reason not to go to a health facility to see a

| 1….. | 2….. | 3….. | 4….. | 5….. |
| --- | --- | --- | --- | --- |
|  |  |  |  |  |
|  |  |  |  |  |
|  |  |  |  |  |
|  |  |  |  |  |
|  |  |  |  |  |
|  |  |  |  |  |

doctor/nurse or not to have an operation or dressings?

No money for health care

No (money for) transportation

No time (person died before arrangements)

Fear / no trust

Not available (facility/personnel/equipment)

No need (condition is not surgical)

**D5. Traditional Healer:**

| 1….. | 2….. | 3….. | 4….. | 5….. |
| --- | --- | --- | --- | --- |
|  |  |  |  |  |
|  |  |  |  |  |

Did the household member go to a traditional healer, traditional doctor,

witch doctor, or religious healer for this problem? Yes No

| 1….. | 2….. | 3….. | 4….. | 5….. |
| --- | --- | --- | --- | --- |
|  |  |  |  |  |
|  |  |  |  |  |
|  |  |  |  |  |

**D6. Location of death:**

Where did the household member die?

Home

Health Facility

Somewhere else

**D7. Give a brief explanation of the story told.**

_____________________________________________________________________________________________________

_____________________________________________________________________________________________________

_____________________________________________________________________________________________________

*[If there were more than one deceased household member, fill in the second or third person’s details in the appropriate spaces]*

| ‘Thank you very much for answering these questions. Let me go quickly over the survey to check everything.’ |
| --- |

*[Go over all the responses to ensure that you have everything, ask the questions again, which you accidentally skipped.]*

**I checked Sections A-D, and there is no data missing:**

*_______________ [date] ______________ [name] _______________ [signature of interviewer]*

| ‘Thank you very much for giving all this information. Can I speak to the household members who I have randomly chosen to ask them more specific questions about their health?’ |
| --- |

*[If the household members are not available now, you should make an appointment for later on that day or the next day.]*

[TIME: ____ hour ____ min]

***THE FOLLOWING QUESTIONS IN SECTION E, F, G, H, I, J, K, and L ARE TO BE ASKED TO THE TWO HOUSEHOLD MEMBERS OF THE FAMILY WHO WERE RANDOMLY SELECTED FOR THE INTERVIEW.***

E. GENERAL INFORMATION [TIME: ____ hour ____ min]

**E1. Household list (ID) number:** *[from the table with the questions B1 and B2]*

_____ *[Check this box if this is a replaced household member]*

**E2. Sex:** *[If a surrogate is answering for a child, ask all the questions about the child.]*

___ Male

___ Female

**E3. Age:**

_________

*[Age for babies:*

*<3 months = 0*

*3- <6 months = 0.25*

*6- <9 months = 0.5*

*9- <12 months = 0.75*

*12-24 months = 1 etc.]*

*[If the household member is 18 or older, then read the following]*

| Good morning/evening. My name is [*Give your name*]. I am a member of the village health team and we are working with Makerere University and the Ministry of Health [*show the information letter*].  We are wondering if you would like to participate in a research study to find out if there are enough doctors in the area, particularly if there are enough surgeons. A surgeon is a medical doctor who cures patients by taking care of wounds and broken bones or cutting out masses. Sometimes surgeons must put you to sleep to do these things, and other times they must only numb the hurt body part.  You were selected as a possible participant because you live in one of the districts we want to learn more about. I want to take a couple minutes to tell you more about this study. Please ask me questions at any time if you do not understand something that I say.  Dr. Moses Galukande, a surgeon from Makerere University, is working with 3 other surgeons: Dr. Samuel Luboga from Makerere University, Dr. Michael Haglund from Duke University in the USA, and Dr. Jeffrey Chipman from the University of Minnesota in the USA. Dr. Mukumbi Fred from Makerere University, who is a specialist in doing studies to learn about an entire population of people, is also working on this study. The study is funded by [*will insert funding once confirmed.].*  We are doing this study to find out what kinds of medical problems are common in Uganda, particularly problems that could be treated by surgery. We want to find out what kinds of things make it difficult for people living in your area to get health care when needed.  We have already asked your family member about some basic information about your household and now we would like to ask you some questions about your health, such as whether you have ever had wounds, broken bones, or masses. The survey will take about 30 minutes. We won’t be offering any medical care right now, but we hope that the information you provide will help bring improved services in the future.  The study has the following risks. We want to make sure that you understand that we are not offering medical care right now. We are not doctors and are not experts in surgery. If we uncover a health problem that you have that might need medical care, we will refer you to the nearest health facility that has the capabilities that you need. I will also ask you to tell me detailed information about your health. You may feel uncomfortable answering some of these questions. I will ask you the questions in a private area where no one will hear your responses except me. I will document your responses on this tablet, but I will never record your name or address. No one will know how you answered any of the questions. If at any time you are too uncomfortable to answer one of the questions, you are welcome to not answer that question or stop the survey altogether.  There are a couple possible direct benefits to you by participating in this study. First, if we do uncover that you have a problem that could be treated by surgery, we can refer you to the closest health facility that can take care of you. Second, by participating in this survey you will learn some information about problems that could possibly be treated by surgery. With this knowledge, you may be able to better recognize if you or one of your family members needs to go to a health center. The main benefit of participating in this survey is that the information you provide can help the Ministry of Health bring more skilled doctors to your area.  The information you provide will be kept private. No information about your identity including your name, address, or birthday will be recorded. The researchers conducting this study and the Uganda Ministry of Health will have access to the information you provide but again, they will not know that it was you who provided the information. In any publications or presentations, the researchers will not include any information that will make it possible to identify you as a study participant. The information you provide will be sent over the internet to the researchers conducting this study, but it will be kept safe with passwords and protections to keep other people from looking at it.  You do not have to participate in the study if you do not want to. If you decide to participate, you are free to stop at any time.  If you have any questions, you may ask them now. If you have questions later, you are encouraged to contact the researchers with the contact information given on the information sheet I gave to your family member. This sheet has all of the information we just talked about. Do you have any questions at the moment? *[Answer any questions they have. If there are questions you cannot answer, contact your Field Supervisor before proceeding.]*  *[Test the subject’s comprehension of the study.]* Now in order to make sure you understand the study, I want to ask you a few simple questions. What are we asking you to do by participating in this study? *[Pause to allow them to answer. Do not go on until you have an adequate answer.]* Why are we asking you questions about your health? *[Pause to allow them to answer. Do not go on until you have an adequate answer.]* |
| --- |

*[If the household member is between 8 and 17, read the following to the parent/guardian]*

| Good morning/evening. My name is [*Give your name*]. I am a member of the village health team and we are working with Makerere University and the Ministry of Health [*show the information letter*].  We are wondering if it is okay for your child to participate in a research study to find out if there are enough doctors in the area, particularly if there are enough surgeons. A surgeon is a medical doctor who cures patients by taking care of wounds and broken bones or cutting out masses. Sometimes surgeons must put you to sleep to do these things, and other times they must only numb the hurt body part.  Your child was selected as a possible participant because you live in one of the districts we want to learn more about. It is important to learn information about people of all ages, which is why we are asking your child to answer the questions. I want to take a couple minutes to tell you more about this study. Please ask me questions at any time if you do not understand something that I say.  Dr. Moses Galukande, a surgeon from Makerere University, is working with 3 other surgeons: Dr. Samuel Luboga from Makerere University, Dr. Michael Haglund from Duke University in the USA, and Dr. Jeffrey Chipman from the University of Minnesota in the USA. Dr. Mukumbi Fred from Makerere University, who is a specialist in doing studies to learn about an entire population of people, is also working on this study. The study is funded by [*will insert funding once confirmed.].*  We are doing this study to find out what kinds of medical problems are common in Uganda, particularly problems that could be treated by surgery. We want to find out what kinds of things make it difficult for people living in your area to get health care when needed.  We have already asked your family member about some basic information about your household and now we would like to ask your child some questions about his/her health, such as whether he/she has ever had wounds, broken bones, or masses. The survey will take about 30 minutes. We won’t be offering any medical care right now, but we hope that the information your child provides will help bring improved services in the future.  The study has the following risks. We want to make sure that you understand that we are not offering medical care right now. We are not doctors and are not experts in surgery. If we uncover a health problem that your child has that might need medical care, we will refer you to the nearest health facility that has the capabilities that he/she needs. I will also ask your child to tell me detailed information about his/her health. He/she will be given the option to answer the questions with or without you present. He/she may feel uncomfortable answering some of these questions. I will ask him/her the questions in a private area where no one will hear the responses except me and you, if your child says it is okay. I will document his/her responses on this tablet, but I will never record his/her name or address. No one will know how he/she answered any of the questions. If at any time he/she is too uncomfortable to answer one of the questions, he/she is welcome to not answer that question or stop the survey altogether.  There are a couple possible direct benefits to your child by participating in this study. First, if we do uncover that he/she has a problem that could be treated by surgery, we can refer him/her to the closest health facility that can take care of him/her. Second, by participating in this survey he/she may learn some information about problems that could possibly be treated by surgery. With this knowledge, he/she may be able to better recognize if he/she or one of your family members needs to go to a health center. The main benefit of participating in this survey is that the information he/she provides can help the Ministry of Health bring more skilled doctors to your area.  The information your child provides will be kept private. No information about his/her identity including his/her name, address, or birthday will be recorded. The researchers conducting this study and the Uganda Ministry of Health will have access to the information he/she provides but again, they will not know that it was your child who provided the information. In any publications or presentations, the researchers will not include any information that will make it possible to identify your child as a study participant. The information your child provides will be sent over the internet to the researchers conducting this study, but it will be kept safe with passwords and protections to keep other people from looking at it.  Your child does not have to participate in the study if you do not want him/her to or if he/she does not want to. If you decide to allow your child to participate, he/she is free to stop at any time.  If you have any questions, you may ask them now. If you have questions later, you are encouraged to contact the researchers with the contact information given on the information sheet I gave to your family member. This sheet has all of the information we just talked about. Do you have any questions at the moment? *[Answer any questions they have. If there are questions you cannot answer, contact your Field Supervisor before proceeding.]*  *[Test the parent’s/guardian’s comprehension of the study.]* Now in order to make sure you understand the study, I want to ask you a few simple questions. What are we asking you to do by participating in this study? *[Pause to allow them to answer. Do not go on until you have an adequate answer.]* Why are we asking you and your child questions about your child’s health? *[Pause to allow them to answer. Do not go on until you have an adequate answer.]* |
| --- |

*[If the household member is between 8 and 17, read the following to the minor]*

| Good morning/evening. My name is [*Give your name*]. We are trying to find out if there are enough doctors in this area, specifically if there are enough surgeons. A surgeon is a medical doctor who cures patients by taking care of wounds and broken bones or cutting out masses. Sometimes surgeons must put you to sleep to do these things, and other times they must only numb the hurt body part. We picked you to participate in the survey because you live in the district we are trying to learn more about. We want to learn about people of all ages, including kids.  To find out if there are enough doctors taking care of these problems in your village, we'd like to ask you some questions. We will ask you questions about your health, such as whether you have ever had wounds, broken bones, or masses. By asking you these questions, we hope that we can help make more skilled doctors available in your village. We won’t be offering medical care right now, but we hope that the information you provide will help create improved services in the future. If we do think you might have a health problem, we will help you find a doctor to take care of you.  This survey will take about 30 minutes. You can choose to answer questions in private without your parent here or you can choose to have your parent here to help you answer questions. You might feel like the questions are very personal. No one will know your answers to the questions except me and your parent, if it is okay with you. You do not have to answer any questions if you don’t want to, and you can stop answering questions at any point. If you have any questions now, you can ask me or if you have any questions later, your parent has the contact information for the researchers. Do you have any questions for me? *[Answer any questions they have. If there are questions you cannot answer, contact your Field Supervisor before proceeding.]* |
| --- |

*[If the household member is less than 8 years old, read the following to the parent]*

| “Good morning/evening. My name is [*Give your name*]. I am a member of the village health team and we are working with Makerere University and the Ministry of Health [*show the information letter*].  We are wondering if it is okay for your child to participate in a research study to find out if there are enough doctors in the area, particularly if there are enough surgeons. A surgeon is a medical doctor who cures patients by taking care of wounds and broken bones or cutting out masses. Sometimes surgeons must put you to sleep to do these things, and other times they must only numb the hurt body part.  Your child was selected as a possible participant because you live in one of the districts we want to learn more about. It is important to learn information about people of all ages, which is why we are asking you questions about your child. I want to take a couple minutes to tell you more about this study. Please ask me questions at any time if you do not understand something that I say.  Dr. Moses Galukande, a surgeon from Makerere University, is working with 3 other surgeons: Dr. Samuel Luboga from Makerere University, Dr. Michael Haglund from Duke University in the USA, and Dr. Jeffrey Chipman from the University of Minnesota in the USA. Dr. Mukumbi Fred from Makerere University, who is a specialist in doing studies to learn about an entire population of people, is also working on this study. The study is funded by [*will insert funding once confirmed.].*  We are doing this study to find out what kinds of medical problems are common in Uganda, particularly problems that could be treated by surgery. We want to find out what kinds of things make it difficult for people living in your area to get health care when needed.  We have already asked your family member about some basic information about your household and now we would like to you some questions about your child’s health, such as whether he/she has ever had wounds, broken bones, or masses. The survey will take about 30 minutes. We won’t be offering any medical care right now, but we hope that the information your child provides will help bring improved services in the future.  The study has the following risks. We want to make sure that you understand that we are not offering medical care right now. We are not doctors and are not experts in surgery. If we uncover a health problem that your child has that might need medical care, we will refer you to the nearest health facility that has the capabilities that he/she needs. I will also ask you to to tell me detailed information about your child’s health. You may feel uncomfortable answering some of these questions. I will ask you the questions in a private area where no one will hear the responses except me. I will document your responses on this tablet, but I will never record your child’s name or address. No one will know how you answered any of the questions. If at any time you are too uncomfortable to answer one of the questions, you is welcome to not answer that question or stop the survey altogether.  There are a couple possible direct benefits to your child by participating in this study. First, if we do uncover that he/she has a problem that could be treated by surgery, we can refer him/her to the closest health facility that can take care of him/her. Second, by participating in this survey you may learn some information about problems that could possibly be treated by surgery. With this knowledge, you may be able to better recognize if you or one of your family members needs to go to a health center. The main benefit of participating in this survey is that the information you provide can help the Ministry of Health bring more skilled doctors to your area.  The information you provide about your child will be kept private. No information about his/her identity including his/her name, address, or birthday will be recorded. The researchers conducting this study and the Uganda Ministry of Health will have access to the information you provide but again, they will not know that it was you who provided the information. In any publications or presentations, the researchers will not include any information that will make it possible to identify your child as a study participant. The information you provide will be sent over the internet to the researchers conducting this study, but it will be kept safe with passwords and protections to keep other people from looking at it.  Your child does not have to participate in the study if you do not want him/her to. If you decide to answer questions about your child, you are free to stop at any time.  If you have any questions, you may ask them now. If you have questions later, you are encouraged to contact the researchers with the contact information given on the information sheet I gave to your family member. This sheet has all of the information we just talked about. Do you have any questions at the moment? *[Answer any questions they have. If there are questions you cannot answer, contact your Field Supervisor before proceeding.]*  *[Test the parent’s/guardian’s comprehension of the study.]* Now in order to make sure you understand the study, I want to ask you a few simple questions. What are we asking you to do by participating in this study? *[Pause to allow them to answer. Do not go on until you have an adequate answer.]* Why are we asking you questions about your child’s health? *[Pause to allow them to answer. Do not go on until you have an adequate answer.]* |
| --- |

*[If the household member is older than 18 years old but has a mental disability and is not able to consent for themselves, read the following to the person giving surrogate consent. You must ask questions to determine if the individual is able to consent for themselves. If they can understand the purpose of the study, the risks and benefits of the study, and what we are asking them to do, then they may consent for themselves. If not, surrogate consent may be given by any relative living in the same household.]*

Good morning/evening. My name is [*Give your name*]. I am a member of the village health team and we are working with Makerere University and the Ministry of Health [*show the information letter*].

We are wondering if it is okay for your relative to participate in a research study to find out if there are enough doctors in the area, particularly if there are enough surgeons. A surgeon is a medical doctor who cures patients by taking care of wounds and broken bones or cutting out masses. Sometimes surgeons must put you to sleep to do these things, and other times they must only numb the hurt body part.

Your relative was selected as a possible participant because you live in one of the districts we want to learn more about. It is important to learn information about people of all abilities, which is why we are asking your permission for your relative to participate in the study. I want to take a couple minutes to tell you more about this study. Please ask me questions at any time if you do not understand something that I say.

Dr. Moses Galukande, a surgeon from Makerere University, is working with 3 other surgeons: Dr. Samuel Luboga from Makerere University, Dr. Michael Haglund from Duke University in the USA, and Dr. Jeffrey Chipman from the University of Minnesota in the USA. Dr. Mukumbi Fred from Makerere University, who is a specialist in doing studies to learn about an entire population of people, is also working on this study. The study is funded by [*will insert funding once confirmed.].*

We are doing this study to find out what kinds of medical problems are common in Uganda, particularly problems that could be treated by surgery. We want to find out what kinds of things make it difficult for people living in your area to get health care when needed.

We have already asked your family member about some basic information about your household and now we would like to ask your relative some questions about his/her health, such as whether he/she has ever had wounds, broken bones, or masses. The survey will take about 30 minutes. We won’t be offering any medical care right now, but we hope that the information your child provides will help bring improved services in the future.

The study has the following risks. We want to make sure that you understand that we are not offering medical care right now. We are not doctors and are not experts in surgery. If we uncover a health problem that your relative has that might need medical care, we will refer you to the nearest health facility that has the capabilities that he/she needs. I will also ask your relative to tell me detailed information about his/her health. He/she will be given the option to answer the questions with or without you present. He/she may feel uncomfortable answering some of these questions. I will ask him/her the questions in a private area where no one will hear the responses except me and you, if your relative says it is okay. I will document his/her responses on this tablet, but I will never record his/her name or address. No one will know how he/she answered any of the questions. If at any time he/she is too uncomfortable to answer one of the questions, he/she is welcome to not answer that question or stop the survey altogether.

There are a couple possible direct benefits to your relative by participating in this study. First, if we do uncover that he/she has a problem that could be treated by surgery, we can refer him/her to the closest health facility that can take care of him/her. Second, by assisting your relative in participating in this survey you may learn some information about problems that could possibly be treated by surgery. With this knowledge, you may be able to better recognize if you or one of your family members needs to go to a health center. The main benefit of participating in this survey is that the information your relative provides can help the Ministry of Health bring more skilled doctors to your area.

The information your relative provides will be kept private. No information about his/her identity including his/her name, address, or birthday will be recorded. The researchers conducting this study and the Uganda Ministry of Health will have access to the information he/she provides but again, they will not know that it was your relative who provided the information. In any publications or presentations, the researchers will not include any information that will make it possible to identify your relative as a study participant. The information your relative provides will be sent over the internet to the researchers conducting this study, but it will be kept safe with passwords and protections to keep other people from looking at it.

Your relative does not have to participate in the study if you do not want him/her to or if he/she does not want to. If you decide to allow your relaive to participate, he/she is free to stop at any time.

If you have any questions, you may ask them now. If you have questions later, you are encouraged to contact the researchers with the contact information given on the information sheet I gave to your family member. This sheet has all of the information we just talked about. Do you have any questions at the moment? *[Answer any questions they have. If there are questions you cannot answer, contact your Field Supervisor before proceeding.]*

*[Test the surrogate’s comprehension of the study.]* Now in order to make sure you understand the study, I want to ask you a few simple questions. What are we asking your relative to do by participating in this study? *[Pause to allow them to answer. Do not go on until you have an adequate answer.]* Why are we asking you and your relative questions about your relative’s health? *[Pause to allow them to answer. Do not go on until you have an adequate answer.]*

*[If the household member is older than 18 years old but has a mental disability and is not able to consent for themselves, read the following to household member to be interviewed]*

| Good morning/evening. My name is [*Give your name*]. We are trying to find out if there are enough doctors in this area, specifically if there are enough surgeons. A surgeon is a medical doctor who cures patients by taking care of wounds and broken bones or cutting out masses. Sometimes surgeons must put you to sleep to do these things, and other times they must only numb the hurt body part. We picked you to participate in the survey because you live in the district we are trying to learn more about.  To find out if there are enough doctors taking care of these problems in your village, we'd like to ask you some questions. We will ask you questions about your health, such as whether you have ever had wounds, broken bones, or masses. By asking you these questions, we hope that we can help make more skilled doctors available in your village. We won’t be offering medical care right now, but we hope that the information you provide will help create improved services in the future. If we do think you might have a health problem, we will help you find a doctor to take care of you.  This survey will take about 30 minutes. You can choose to answer questions in private without your relative here or you can choose to have your relative here to help you answer questions. You might feel like the questions are very personal. No one will know your answers to the questions except me and your relative, if it is okay with you. You do not have to answer any questions if you don’t want to, and you can stop answering questions at any point. If you have any questions now, you can ask me or if you have any questions later, your relative has the contact information for the researchers. Do you have any questions for me? *[Answer any questions they have. If there are questions you cannot answer, contact your Field Supervisor before proceeding.]* |
| --- |

**E4. Informed consent:**

Do you understand what we have talked about and have you had all of your questions answered?

Do yu consent to participation in this study? OR Is it okay for your son/daughter/relative to participate in this survey?

___ Yes

___ No, what is the reason? (no time / no willingness / no reason / no seen benefit / other: explain….)

*[For minors (individuals under age 18), this consent E4 is obtained from a guardian/parent. Without informed consent you cannot proceed. Make sure the person understands the purpose of this survey. If they don’t want to participate, ask why and mark this.]*

**E5. Assent:** *[children between 8 and 17 years of age and adults who cannot consent for themselves only]*

Would you like to participate in this survey?

___ Yes

___ No, what is the reason? (no time / no willingness / no reason / no seen benefit / other: explain….)

*[If a child under the age of 8 years is not around for the interview, a parent/guardian may answer the questions for the child. For children between 8 and 17 years, both parent/guardian consent and minor assent must be obtained before proceeding. The minor may choose to answer the questions with or without the help of a parent/guardian. For adults with limited mental capacity, participant assent and surrogate consent by a relative must be obtained.]*

| The following questions are general questions. Later on I will ask more about your health. |
| --- |

**E6. Education:**

What is the highest educational level that you have achieved or are currently pursuing?

___ None (includes nursery)

___ Primary school

___ Secondary school (junior / senior)

___ Technical school

___ Tertiary (diploma, university, bachelors)

___ Graduate degree (Master degree, PhD)

**E6.1 Literacy:**

Are you able to read and write in any language?

*[For adults and children who are currently learning how to read and write answer: ‘No’]*

*_*__ Yes

___ No

**E7. Occupation:**

What is your primary occupation?

___ Unemployed *[Currently looking for jobs, retiree’s]*

___ Student [*Primary, Secondary, University, or Graduate Program*]

___ Homemaker *[Housewives]*

___ Domestic helpers *[Cleaners, housekeepers, watch guards]*

___ Farmer *[Herders, agriculture, pastoralist]*

___ Self-employed / small-business *[Small business owners like: shops, kiosks, food traders]*

___ Government employee *[Professional working for government agency, police officer, accountant,
 teachers, health care workers, etc.]*

___ Non-government employee *[Professional working for NGO, corporation, bank, etc.]*

**E8. Ethnic Group?**

What is your ethnic background? *[In case of refusal to answer, ask if the person was born in the Ugandan Region]*

| _____ Acholi  _____ Alur  _____ Aliba  _____ Aringa  _____ Baamba  _____ Babukusu  _____ Babwisi  _____ Bafumbira  _____ Baganda  _____ Bagisu  _____ Bagungu  _____ Bagwe  _____ Bagwere  _____ Bahehe  _____ Bahororo  _____ Bakenyi  _____ Bakiga  _____ Bakonzo  _____ Banyabindi  _____ Banyabutumbi  _____ Banyankore  _____ Banyara  _____ Banyaruguru  _____ Banyarwanda  _____ Banyole  _____ Baruli  _____ Barundi  _____ Basamia  _____ Basoga  _____ Basongora  _____ Batagwenda  _____ Batoro  _____ Batuku  _____ Batwa | _____ Chope  _____ Dodoth  _____ Ehtur  _____ Gimara  _____ Ik (Teuso)  _____ Iteso  _____ Jie  _____ Jonam  _____ Jopadhola  _____ Kakwa  _____ Karamojong  _____ Kebu (Okebu)  _____ Kuku  _____ Kuman  _____ Langi  _____ Lendu  _____ Lugbara  _____ Madi  _____ Mening  _____ Mvuba  _____ Napore  _____ Ngikutio  _____ Nubi  _____ Nyangia  _____ Pokot  _____ Reli  _____ Sabiny  _____ Shana  _____ So (Tepeth)  _____ Vonoma  _____ Other Ugandan  _____ Other African  _____ Non African |
| --- | --- |

**E9. Religion:**

What is your religion?

_____ Catholic

_____ Protestant

_____ Other Christian

_____ Muslim

_____ Traditional

_____ Other

_____ None

**E10. Length of stay in house:**

How many years have you lived in this household?

_____ *[years]*

**E11. Health status:**

Are you generally healthy?

___ Yes

___ No

*[if ‘Yes’ to E11, skip questions E12, E13 and E14]*

**E12. Time ill:**

In total how many weeks have you been ill during the past year?

_____ *[weeks]*

**E13. Number of health facility visits:**

How many times have you visited a clinic or hospital, or nurse / medical doctor in the last year?

_____

**E14. Recovery from illness:**

Have you recovered fully from the illness you had?

___ Yes

___ No

*[EXPLAIN:]*

| Surgery, also known as an operation, can be done for a swelling, mass, abdominal pain, and many other things. Patients often have a bandage after having surgery or may need to stay in the hospital for some time. Sometimes, children are born with problems that can be fixed with an operation. Examples of these problems are open lips, missing anus, or strange feet.  Some people who break a bone or have a wound, may not have an operation but still need to be seen by a doctor or stay in the surgical ward of a hospital. Since this does not include an operation, but includes surgical consultation, we call it surgical care.  Now I'm going to ask you about all the surgical problems you've had in your lifetime. We'll start with your head and move all the way down to your toes. |
| --- |

[TIME: ____ hour ____ min]

F. FACE / HEAD / NECK

**F1.1 Face / head / neck:**

Have you ever had a wound, burn, infection that needed to be drained or opened, mass / goiter, deformity, problem with eating/drinking, a problem with your eyes or ears or an operation on your face, head, or neck?

___ Yes

___ No

*[If there were/are no problems with this anatomical section you can continue with section G. If the person had a problem with this anatomical section, answer all the questions for problem 1 before moving on to any additional problems.]*

**F1.2 Face / head / neck location:**

| Problem 1 | Problem 2 | Problem 3 |
| --- | --- | --- |
|  |  |  |
|  |  |  |
|  |  |  |
|  |  |  |
|  |  |  |

On what part of your head / neck / face did the problem occur?

Eye

Ear / nose / throat

Dental / lips / mouth

Neck

Head

**F2.1 Face / head / neck specifics:**

| Problem 1 | Problem 2 | Problem 3 |
| --- | --- | --- |
|  |  |  |
|  |  |  |
|  |  |  |
|  |  |  |
|  |  |  |
|  |  |  |

Tell me what problem you have had.

Wound or infection injury related

Wound or infection not injury related

Burn

Mass or growth / goiter

Deformity congenital

Deformity acquired

*[Wound: Open skin; sometimes leaking blood, pus or liquid*

*Deformity: An abnormal tissue arrangement or malformation*

*Congenital: The person is born with the problem. Think about: cleft lips, hydrocephalus etc.*

*Acquired: The person got the problem later in life. Think about: scars and broken bones]*

**F2.2 Type of injury / accident:**

Did the problem start after an injury or accident? What kind of accident?

| Problem 1 | Problem 2 | Problem 3 |
| --- | --- | --- |
|  |  |  |
|  |  |  |
|  |  |  |
|  |  |  |
|  |  |  |
|  |  |  |
|  |  |  |
|  |  |  |
|  |  |  |
|  |  |  |

*[Pick the one that best describes the injury / accident. Pedestrian and bicycle crash definition: there was no motorized vehicle involved. All can be intentional or unintentional.]*

No, it was not due to an injury / accident

Car, truck, bus crash

Motorcycle crash

Pedestrian, bicycle crash

Gunshot

Stab / slash / cut / crush

Bite or animal attack

Fall

Open fire / explosion

Hot liquid / hot object

| Problem 1 | Problem 2 | Problem 3 |
| --- | --- | --- |
|  |  |  |
|  |  |  |
|  |  |  |

**F3.1 Timing:**

When did this problem start? In the last month

During the past 12 months but longer than a month ago

Longer than 12 months ago

| Problem 1 | Problem 2 | Problem 3 |
| --- | --- | --- |
|  |  |  |
|  |  |  |

**F3.2 At this moment:**

Do you have this problem now (or during the last week)? Yes No

| Problem 1 | Problem 2 | Problem 3 |
| --- | --- | --- |
|  |  |  |
|  |  |  |

**F4.1 Healthcare sought:**

Did you go to a health facility or see a doctor/nurse for this problem? Yes No

**F4.2 Type of healthcare received:** *[Only when ‘yes’ to F4.1]*

What kind of treatment did you receive?

*[A major procedure is a procedure which requires regional/spinal/general anesthesia; A minor procedures is a procedure requiring local anaesthesia, dressings, wound care, punctures, suturing or incision and drainage]*

| Problem 1 | Problem 2 | Problem 3 |
| --- | --- | --- |
|  |  |  |
|  |  |  |
|  |  |  |

None / No surgical care

Major procedure

Minor procedures

**F4.3 Reason for not having surgical care:** *[Only when ‘none / no surgical care’ to F4.2 or ‘no’ to F4.1]*

What was the main reason not to go to a health facility to see a doctor/nurse or not to have an operation or dressings?

*[Select all the answers that are true. If the person was referred but did not go to the referral hospital, mark the answer why (s)he did not go here.]*

| Problem 1 | Problem 2 | Problem 3 |
| --- | --- | --- |
|  |  |  |
|  |  |  |
|  |  |  |
|  |  |  |
|  |  |  |
|  |  |  |

No money for health care

No (money for) transportation

No time

Fear / no trust

Not available (facility/personnel/equipment)

No need

| Problem 1 | Problem 2 | Problem 3 |
| --- | --- | --- |
|  |  |  |
|  |  |  |

**F5. Traditional Healer:**

Did you go to a traditional healer, traditional doctor, witch doctor Yes

or bone setter for this problem? No

**F6. Disability:**

| Problem 1 | Problem 2 | Problem 3 |
| --- | --- | --- |
|  |  |  |
|  |  |  |
|  |  |  |
|  |  |  |
|  |  |  |

Does this problem still impact your daily life?

The condition is not disabling

I feel ashamed

I'm not able to work like I used to

I need help with transportation

I need help with daily living

*[Disability: a physical problem that impacts your life, or makes it difficult to carry out your daily activities. Select the highest level of disability the person experiences. Select only one answer.]*

*[Ask if the person had another problem in this anatomical location. If so, go to the following column (problem 2 or 3) to register this problem. If there are no other problems in this location, go to the following anatomical location. In case of more than 3 problems, mark the ones which are most recent and most relevant for the respondent.]*

G. CHEST

**G1. Chest:**

Have you ever had a wound, burn, infection that needed to be drained or opened, deformity, or an operation on your chest (including heart or lungs)?

___ Yes

___ No

*[If there were/are no problems with this anatomical section you can continue with section H. If the person had a problem with this anatomical section, answer all the questions for problem 1 before moving on to any additional problems.]*

**G2.1. Chest specifics:**

| Problem 1 | Problem 2 | Problem 3 |
| --- | --- | --- |
|  |  |  |
|  |  |  |
|  |  |  |
|  |  |  |
|  |  |  |
|  |  |  |

Tell me what problem you have had.

Wound or infection injury related

Wound or infection not injury related

Burn

Lung cancer

Deformity congenital

Deformity acquired

*[Wound: Open skin; sometimes leaking blood, pus or liquid*

*Deformity: An abnormal tissue arrangement, malformation*

*Congenital: The person is born with the problem. Example: heart malformation*

*Acquired: The person got the problem later in life. Example: problem with one of the heart valves.]*

**G2.2 Type of injury / accident:**

Did the problem start after an injury or accident? What kind of accident?

| Problem 1 | Problem 2 | Problem 3 |
| --- | --- | --- |
|  |  |  |
|  |  |  |
|  |  |  |
|  |  |  |
|  |  |  |
|  |  |  |
|  |  |  |
|  |  |  |
|  |  |  |
|  |  |  |

*[Pick the one that best describes the injury / accident. Pedestrian and bicycle crash definition: there was no motorized vehicle involved. All can be intentional or unintentional.]*

No, it was not due to an injury / accident

Car, truck, bus crash

Motorcycle crash

Pedestrian, bicycle crash

Gunshot

Stab / slash / cut / crush

Bite or animal attack

Fall

Open fire / explosion

Hot liquid / hot object

| Problem 1 | Problem 2 | Problem 3 |
| --- | --- | --- |
|  |  |  |
|  |  |  |
|  |  |  |

**G3.1. Timing:**

When did this problem start?

In the last month

During the past 12 months but longer than a month ago

Longer than 12 months ago

| Problem 1 | Problem 2 | Problem 3 |
| --- | --- | --- |
|  |  |  |
|  |  |  |

**G3.2 At this moment:**

Do you have this problem now (or during the last week)? Yes No

| Problem 1 | Problem 2 | Problem 3 |
| --- | --- | --- |
|  |  |  |
|  |  |  |

**G4.1 Healthcare sought:**

Did you go to a health facility or see a doctor/nurse for this problem? Yes No

**G4.2 Type of healthcare received:** *[Only when ‘yes’ to G4.1]*

What kind of treatment did you receive?

*[A major procedure is a procedure which requires regional/spinal/general anesthesia; A minor procedures is a procedure requiring local anaesthesia, dressings, wound care, punctures, suturing or incision and drainage]*

| Problem 1 | Problem 2 | Problem 3 |
| --- | --- | --- |
|  |  |  |
|  |  |  |
|  |  |  |

None / No surgical care

Major procedure

Minor procedures

**G4.3 Reason for not having surgical care:** *[Only when ‘none / no surgical care’ to G4.2 or ‘no’ to G4.1]*

What was the main reason not to go to a health facility to see a doctor/nurse or not to have an operation or dressings?

*[Select all the answers that are true. If the person was referred but did not go to the referral hospital, mark the answer why (s)he did not go here.]*

| Problem 1 | Problem 2 | Problem 3 |
| --- | --- | --- |
|  |  |  |
|  |  |  |
|  |  |  |
|  |  |  |
|  |  |  |
|  |  |  |

No money for health care

No (money for) transportation

No time

Fear / no trust

Not available (facility/personnel/equipment)

No need

| Problem 1 | Problem 2 | Problem 3 |
| --- | --- | --- |
|  |  |  |
|  |  |  |

**G5. Traditional Healer:**

Did you go to a traditional healer, traditional doctor, witch doctor Yes

or bone setter for this problem? No

**G6. Disability:**

| Problem 1 | Problem 2 | Problem 3 |
| --- | --- | --- |
|  |  |  |
|  |  |  |
|  |  |  |
|  |  |  |
|  |  |  |

Does this problem still impact your daily life?

The condition is not disabling

I feel ashamed

I'm not able to work like I used to

I need help with transportation

I need help with daily living

*[Disability: a physical problem that impacts your life, or makes it difficult to carry out your daily activities. Select the highest level of disability the person experiences. Select only one answer.]*

*[Ask if the person had another problem in this anatomical location. If so, go to the following column (problem 2 or 3) to register this problem. If there are no other problems in this location, go to the following anatomical location. In case of more than 3 problems, mark the ones which are most recent and most relevant for the respondent.]*

H. BACK

**H1. Back:**

Have you ever had a wound, burn, infection that needed to be drained or opened, mass, deformity, or an operation on your back? Have you had severe back pain that lasted longer than 6 weeks, that had shooting pain into your arms or legs, or that was accompanied with numbness or tingling in your arms or legs?

___ Yes

___ No

*[If there were/are no problems with this anatomical section you can continue with section I. If the person had a problem with this anatomical section, answer all the questions for problem 1 before moving on to any additional problems.]*

**H2.1 Back specifics:**

| Problem 1 | Problem 2 | Problem 3 |
| --- | --- | --- |
|  |  |  |
|  |  |  |
|  |  |  |
|  |  |  |
|  |  |  |
|  |  |  |
|  |  |  |

Tell me what problem you have had.

Wound or injury injury related

Wound or injury not injury related

Burn

Mass or growth

Deformity congenital

Deformity acquired

Back pain

*[Wound: Open skin; sometimes leaking blood, pus or liquid*

*Deformity: An abnormal tissue arrangement, malformation*

*Congenital: The person is born with the problem.*

*Acquired: The person got the problem later in life*

*Back Pain: Must be lasting >6 weeks, accompanied by shooting pain in the extremities, or accompanied by numbness or tingling in the extremities. ]*

**H2.2 Type of injury / accident:**

Did the problem start after an injury or accident? What kind of accident?

| Problem 1 | Problem 2 | Problem 3 |
| --- | --- | --- |
|  |  |  |
|  |  |  |
|  |  |  |
|  |  |  |
|  |  |  |
|  |  |  |
|  |  |  |
|  |  |  |
|  |  |  |
|  |  |  |

*[Pick the one that best describes the injury / accident. Pedestrian and bicycle crash definition: there was no motorized vehicle involved. All can be intentional or unintentional.]*

No, it was not due to an injury / accident

Car, truck, bus crash

Motorcycle crash

Pedestrian, bicycle crash

Gunshot

Stab / slash / cut / crush

Bite or animal attack

Fall

Open fire / explosion

Hot liquid / hot object

| Problem 1 | Problem 2 | Problem 3 |
| --- | --- | --- |
|  |  |  |
|  |  |  |
|  |  |  |

**H3.1 Timing:**

When did this problem start? In the last month

During the past 12 months but longer than a month ago

Longer than 12 months ago

**H3.2 At this moment:**

| Problem 1 | Problem 2 | Problem 3 |
| --- | --- | --- |
|  |  |  |
|  |  |  |

Do you have this problem now (or during the last week)? Yes No

| Problem 1 | Problem 2 | Problem 3 |
| --- | --- | --- |
|  |  |  |
|  |  |  |

**H4.1 Healthcare sought:**

Did you go to a health facility or see a doctor/nurse for this problem? Yes No

**H4.2 Type of healthcare received:** *[Only when ‘yes’ to H4.1]*

What kind of treatment did you receive?

*[A major procedure is a procedure which requires regional/spinal/general anesthesia; A minor procedures is a procedure requiring local anaesthesia, dressings, wound care, punctures, suturing or incision and drainage]*

| Problem 1 | Problem 2 | Problem 3 |
| --- | --- | --- |
|  |  |  |
|  |  |  |
|  |  |  |

None / No surgical care

Major procedure

Minor procedures

**H4.3 Reason for not having surgical care:** *[Only when ‘none / no surgical care’ to H4.2 or ‘no’ to H4.1]*

What was the main reason not to go to a health facility to see a doctor/nurse or not to have an operation or dressings?

*[Select all the answers that are true. If the person was referred but did not go to the referral hospital, mark the answer why (s)he did not go here.]*

| Problem 1 | Problem 2 | Problem 3 |
| --- | --- | --- |
|  |  |  |
|  |  |  |
|  |  |  |
|  |  |  |
|  |  |  |
|  |  |  |

No money for health care

No (money for) transportation

No time

Fear / no trust

Not available (facility/personnel/equipment)

No need

| Problem 1 | Problem 2 | Problem 3 |
| --- | --- | --- |
|  |  |  |
|  |  |  |

**H5. Traditional Healer:**

Did you go to a traditional healer, traditional doctor, witch doctor Yes

or bone setter for this problem? No

**H6. Disability:**

| Problem 1 | Problem 2 | Problem 3 |
| --- | --- | --- |
|  |  |  |
|  |  |  |
|  |  |  |
|  |  |  |
|  |  |  |

Does this problem still impact your daily life?

The condition is not disabling

I feel ashamed

I'm not able to work like I used to

I need help with transportation

I need help with daily living

*[Disability: a physical problem that impacts your life, or makes it difficult to carry out your daily activities. Select the highest level of disability the person experiences. Select only one answer.]*

*[Ask if the person had another problem in this anatomical location. If so, go to the following column (problem 2 or 3) to register this problem. If there are no other problems in this location, go to the following anatomical location. In case of more than 3 problems, mark the ones which are most recent and most relevant for the respondent.]*

I. ABDOMEN

**I1. Abdomen:**

Have you ever had a wound, burn, infection that needed to be drained or opened, mass, cancer, or deformity in or on your abdomen or had severe abdominal pain or bloating, trouble having a baby (obstructed labor), an abdominal delivery (Cessarian), or any other operation on your abdomen?

___ Yes

___ No

*[If there were/are no problems with this anatomical section you can continue with section J. If the person had a problem with this anatomical section, answer all the questions for problem 1 before moving on to any additional problems.]*

**I2.1. Abdomen specifics:**

| Problem 1 | Problem 2 | Problem 3 |
| --- | --- | --- |
|  |  |  |
|  |  |  |
|  |  |  |
|  |  |  |
|  |  |  |
|  |  |  |
|  |  |  |
|  |  |  |
|  |  |  |
|  |  |  |

Tell me what problem you have had.

Wound or infection injury related

Wound or infection not injury related

Burn

Mass, growth, or cancer (solid)

Hernia (Mass or growth that is soft and reducible,)

Deformity congenital

Deformity acquired

Abdominal pain (such as from gall stones or appendicitis)

Abdominal Distention

Obstructed labor or Cessarian section

**I2.2 Type of injury / accident:**

Did the problem start after an injury or accident? What kind of accident?

| Problem 1 | Problem 2 | Problem 3 |
| --- | --- | --- |
|  |  |  |
|  |  |  |
|  |  |  |
|  |  |  |
|  |  |  |
|  |  |  |
|  |  |  |
|  |  |  |
|  |  |  |
|  |  |  |

*[Pick the one that best describes the injury / accident. Pedestrian and bicycle crash definition: there was no motorized vehicle involved. All can be intentional or unintentional.]*

No, it was not due to an injury / accident

Car, truck, bus crash

Motorcycle crash

Pedestrian, bicycle crash

Gunshot

Stab / slash / cut / crush

Bite or animal attack

Fall

Open fire / explosion

Hot liquid / hot object

**I2.3 Abdominal Pain Location:** *[only answer when answer to I2.1 is “abdominal pain”]*

Where was the pain predominantly located?

| Problem 1 | Problem 2 | Problem 3 |
| --- | --- | --- |
|  |  |  |
|  |  |  |
|  |  |  |
|  |  |  |
|  |  |  |
|  |  |  |
|  |  |  |

Left upper quadrant

Right Upper Quadrant

Left lower quadrant

Right lower quadrant

Peri-umbilical

Epigastrium

Suprapubic

*[Periumbilical: pain surrounding the umbilicus*

*Epigastrium: pain in the upper middle of the abdomen*

*Suprapubic: pain in the lower middle of the abdomen]*

**I2.4 Abdominal Pain Timing:** *[only answer when answer to I2.1 is “abdominal pain”]*

For how long did you have the pain?

| Problem 1 | Problem 2 | Problem 3 |
| --- | --- | --- |
|  |  |  |
|  |  |  |
|  |  |  |
|  |  |  |
|  |  |  |
|  |  |  |
|  |  |  |

Less than 1 day

Between 1 and 3 days

4 to 6 days

1 week to 1 month

1 month to 6 months

6 months to 1 year

Greater than 1 year

**I2.5 Abdominal Distention Timing:** *[only answer when answer to I2.1 is “abdominal distention”]*

For how long did you have the bloating/distention?

| Problem 1 | Problem 2 | Problem 3 |
| --- | --- | --- |
|  |  |  |
|  |  |  |
|  |  |  |
|  |  |  |
|  |  |  |
|  |  |  |
|  |  |  |

Less than 1 day

Between 1 and 3 days

4 to 6 days

1 week to 1 month

1 month to 6 months

6 months to 1 year

Greater than 1 year

| Problem 1 | Problem 2 | Problem 3 |
| --- | --- | --- |
|  |  |  |
|  |  |  |
|  |  |  |

**I3.1 Timing:**

When did this problem start? In the last month

During the past 12 months but longer than a month ago

Longer than 12 months ago

| Problem 1 | Problem 2 | Problem 3 |
| --- | --- | --- |
|  |  |  |
|  |  |  |

**I3.2 At this moment:**

Do you have this problem now (or during the last week)? Yes No

| Problem 1 | Problem 2 | Problem 3 |
| --- | --- | --- |
|  |  |  |
|  |  |  |

**I4.1 Healthcare sought:**

Did you go to a health facility or see a doctor/nurse for this problem?

Yes

No

**I4.2 Type of healthcare received:** *[Only when ‘yes’ to I4.1]*

What kind of treatment did you receive?

*[A major procedure is a procedure which requires regional/spinal/general anesthesia; A minor procedures is a procedure requiring local anaesthesia, dressings, wound care, punctures, suturing or incision and drainage]*

| Problem 1 | Problem 2 | Problem 3 |
| --- | --- | --- |
|  |  |  |
|  |  |  |
|  |  |  |

None / No surgical care

Major procedure

Minor procedures

**I4.3 Reason for not having surgical care:** *[Only when ‘none / no surgical care’ to I4.2 or ‘no’ to I4.1]*

What was the main reason not to go to a health facility to see a doctor/nurse or not to have an operation or dressings?

*[Select all the answers that are true. If the person was referred but did not go to the referral hospital, mark the answer why (s)he did not go here.]*

| Problem 1 | Problem 2 | Problem 3 |
| --- | --- | --- |
|  |  |  |
|  |  |  |
|  |  |  |
|  |  |  |
|  |  |  |
|  |  |  |

No money for health care

No (money for) transportation

No time

Fear / no trust

Not available (facility/personnel/equipment)

No need

| Problem 1 | Problem 2 | Problem 3 |
| --- | --- | --- |
|  |  |  |
|  |  |  |

**I5. Traditional Healer:**

Did you go to a traditional healer, traditional doctor, witch doctor Yes

or bone setter for this problem? No

**I6. Disability:**

| Problem 1 | Problem 2 | Problem 3 |
| --- | --- | --- |
|  |  |  |
|  |  |  |
|  |  |  |
|  |  |  |
|  |  |  |

Does this problem still impact your daily life?

The condition is not disabling

I feel ashamed

I'm not able to work like I used to

I need help with transportation

I need help with daily living

*[Disability: a physical problem that impacts your life, or makes it difficult to carry out your daily activities. Select the highest level of disability the person experiences. Select only one answer.]*

*[Ask if the person had another problem in this anatomical location. If so, go to the following column (problem 2 or 3) to register this problem. If there are no other problems in this location, go to the following anatomical location. In case of more than 3 problems, mark the ones which are most recent and most relevant for the respondent.]*

J. GROIN / GENITALIA / BUTTOCKS

**J1. Groin / genitalia / buttocks:**

Have you ever had a wound, burn, infection that needed to be drained or opened, mass, deformity, leaking of urine or feces, bleeding from your bottom, bleeding from your penis, or an operation on your groin, genitalia or buttocks?

___ Yes

___ No

*[If there were/are no problems with this anatomical section you can continue with section K. If the person had a problem with this anatomical section, answer all the questions for problem 1 before moving on to any additional problems.]*

| Problem 1 | Problem 2 | Problem 3 |
| --- | --- | --- |
|  |  |  |
|  |  |  |
|  |  |  |
|  |  |  |
|  |  |  |
|  |  |  |
|  |  |  |
|  |  |  |
|  |  |  |
|  |  |  |
|  |  |  |

**J2.1. Groin / genitalia / Buttocks specifics:**

Tell me what problem you have had. Wound or infection due to an injury

Wound or infection not due to an injury

Burn

Mass or growth (solid) *[testicular cancer or hydrocele/cystocele]*

Inguinal Hernia (Mass that is soft or reducible)

Deformity congenital

Deformity acquired

Leaking of urine or feces (like fistula)

Inability to urinate

Bleeding (per rectum)

Bleeding (from the penis)

*[Deformity: An abnormal tissue arrangement, malformation*

*Congenital: The person is born with the problem. Think about: born without anus, hypospadias etc.*

*Acquired: The person got the problem later in life]*

**J2.2 Type of injury / accident:**

Did the problem start after an injury or accident? What kind of accident?

| Problem 1 | Problem 2 | Problem 3 |
| --- | --- | --- |
|  |  |  |
|  |  |  |
|  |  |  |
|  |  |  |
|  |  |  |
|  |  |  |
|  |  |  |
|  |  |  |
|  |  |  |
|  |  |  |

*[Pick the one that best describes the injury / accident. Pedestrian and bicycle crash definition: there was no motorized vehicle involved. All can be intentional or unintentional.]*

No, it was not due to an injury / accident

Car, truck, bus crash

Motorcycle crash

Pedestrian, bicycle crash

Gunshot

Stab / slash / cut / crush

Bite or animal attack

Fall

Open fire / explosion

Hot liquid / hot object

| Problem 1 | Problem 2 | Problem 3 |
| --- | --- | --- |
|  |  |  |
|  |  |  |
|  |  |  |

**J3.1. Timing:**

When did this problem start? In the last month

During the past 12 months but longer than a month ago

Longer than 12 months ago

**J3.2 At this moment:**

| Problem 1 | Problem 2 | Problem 3 |
| --- | --- | --- |
|  |  |  |
|  |  |  |

Do you have this problem now (or during the last week)? Yes No

| Problem 1 | Problem 2 | Problem 3 |
| --- | --- | --- |
|  |  |  |
|  |  |  |

**J4.1 Healthcare sought:**

Did you go to a health facility or see a doctor/nurse for this problem? Yes No

**J4.2 Type of healthcare received:** *[Only when ‘yes’ to J4.1]*

What kind of treatment did you receive?

*[A major procedure is a procedure which requires regional/spinal/general anesthesia; A minor procedures is a procedure requiring local anaesthesia, dressings, wound care, punctures, suturing or incision and drainage]*

| Problem 1 | Problem 2 | Problem 3 |
| --- | --- | --- |
|  |  |  |
|  |  |  |
|  |  |  |

None / No surgical care

Major procedure

Minor procedures

**J4.3 Reason for not having surgical care:** *[Only when ‘none / no surgical care’ to J4.2 or ‘no’ to J4.1]*

What was the main reason not to go to a health facility to see a doctor/nurse or not to have an operation or dressings?

*[Select all the answers that are true. If the person was referred but did not go to the referral hospital, mark the answer why (s)he did not go here.]*

| Problem 1 | Problem 2 | Problem 3 |
| --- | --- | --- |
|  |  |  |
|  |  |  |
|  |  |  |
|  |  |  |
|  |  |  |
|  |  |  |

No money for health care

No (money for) transportation

No time

Fear / no trust

Not available (facility/personnel/equipment)

No need

| Problem 1 | Problem 2 | Problem 3 |
| --- | --- | --- |
|  |  |  |
|  |  |  |

**J5. Traditional Healer:**

Did you go to a traditional healer, traditional doctor, witch doctor Yes

or bone setter for this problem? No

**J6. Disability:**

| Problem 1 | Problem 2 | Problem 3 |
| --- | --- | --- |
|  |  |  |
|  |  |  |
|  |  |  |
|  |  |  |
|  |  |  |

Does this problem still impact your daily life?

The condition is not disabling

I feel ashamed

I'm not able to work like I used to

I need help with transportation

I need help with daily living

*[Disability: a physical problem that impacts your life, or makes it difficult to carry out your daily activities. Select the highest level of disability the person experiences. Select only one answer.]*

*[Ask if the person had another problem in this anatomical location. If so, go to the following column (problem 2 or 3) to register this problem. If there are no other problems in this location, go to the following anatomical location. In case of more than 3 problems, mark the ones which are most recent and most relevant for the respondent.]*

K. EXTREMITIES

**K1.1 Extremities:**

Have you ever had an injury, burn, infection that needed to be drained or opened, wound, mass, deformity, broken bone, or an operation on your hands, feet, arms, or legs?

___ Yes

___ No

*[If there were/are no problems with this anatomical section you can continue with section L. If the person had a problem with this anatomical section, answer all the questions for problem 1 before moving on to any additional problems.]*

| Problem 1 | Problem 2 | Problem 3 |
| --- | --- | --- |
|  |  |  |
|  |  |  |
|  |  |  |
|  |  |  |
|  |  |  |
|  |  |  |
|  |  |  |

**K1.2 Extremity location:**

On what part of your body did the problem occur? Finger(s)

Thumb / Hand

Lower arm

Upper arm

Foot

Lower leg

Upper leg

*[If the problem is right and left: make two separate problems to document the problem.]*

*[If the problem is based on a joint you need to choose the most proximal location to the abdomen.*

*For example: an elbow fracture is documented as the upper arm, a knee skin contracture is documented as the upper leg.]*

**K2.1. Extremity specifics:**

| Problem 1 | Problem 2 | Problem 3 |
| --- | --- | --- |
|  |  |  |
|  |  |  |
|  |  |  |
|  |  |  |
|  |  |  |
|  |  |  |
|  |  |  |

Tell me what problem you have had.

Wound or infection injury related

Wound or infection not injury related

(Recurrent/repeated) drainage / discharge

Burn

Mass or growth

Deformity congenital

Deformity acquired

*[(Recurrent/repeated) drainage / discharge from small sore or opening in the skin: a high suspicion for osteomyelitis, infected bone*

*Deformity: An abnormal tissue arrangement, malformation*

*Congenital: The person is born with the problem. Think about: clubfeet*

*Acquired: The person got the problem later in life. Think about: broken bones and scar contractures]*

**K2.2 Type of injury / accident:**

Did the problem start after an injury or accident? What kind of accident?

| Problem 1 | Problem 2 | Problem 3 |
| --- | --- | --- |
|  |  |  |
|  |  |  |
|  |  |  |
|  |  |  |
|  |  |  |
|  |  |  |
|  |  |  |
|  |  |  |
|  |  |  |
|  |  |  |

*[Pick the one that best describes the injury / accident. Pedestrian and bicycle crash definition: there was no motorized vehicle involved. All can be intentional or unintentional.]*

No, it was not due to an injury / accident

Car, truck, bus crash

Motorcycle crash

Pedestrian, bicycle crash

Gunshot

Stab / slash / cut / crush

Bite or animal attack

Fall

Open fire / explosion

Hot liquid / hot object

| Problem 1 | Problem 2 | Problem 3 |
| --- | --- | --- |
|  |  |  |
|  |  |  |

**K2.3. Fracture:**

Did you break a bone or dislocate a joint? Yes

No

**K3.1. Timing:**

| Problem 1 | Problem 2 | Problem 3 |
| --- | --- | --- |
|  |  |  |
|  |  |  |
|  |  |  |

When did this problem start?

In the last month

During the past 12 months but longer than a month ago

Longer than 12 months ago

| Problem 1 | Problem 2 | Problem 3 |
| --- | --- | --- |
|  |  |  |
|  |  |  |

**K3.2 At this moment:**

Do you have this problem now (or during the last week)? Yes No

| Problem 1 | Problem 2 | Problem 3 |
| --- | --- | --- |
|  |  |  |
|  |  |  |

**K4.1 Healthcare sought:**

Did you go to a health facility or see a doctor/nurse for this problem? Yes No

**K4.2 Type of healthcare received:** *[Only when ‘yes’ to K5.1]*

What kind of treatment did you receive?

*[A major procedure is a procedure which requires regional/spinal/general anesthesia; A minor procedures is a procedure requiring local anaesthesia, dressings, wound care, punctures, suturing or incision and drainage]*

| Problem 1 | Problem 2 | Problem 3 |
| --- | --- | --- |
|  |  |  |
|  |  |  |
|  |  |  |

None / No surgical care

Major procedure

Minor procedures

**K4.3 Reason for not having surgical care:** *[Only when ‘none / no surgical care’ to K5.2 or ‘no’ to K5.1]*

What was the main reason not to go to a health facility to see a doctor/nurse or not to have an operation or dressings?

*[Select all the answers that are true. If the person was referred but did not go to the referral hospital, mark the answer why (s)he did not go here.]*

| Problem 1 | Problem 2 | Problem 3 |
| --- | --- | --- |
|  |  |  |
|  |  |  |
|  |  |  |
|  |  |  |
|  |  |  |
|  |  |  |

No money for health care

No (money for) transportation

No time

Fear / no trust

Not available (facility/personnel/equipment)

No need

| Problem 1 | Problem 2 | Problem 3 |
| --- | --- | --- |
|  |  |  |
|  |  |  |

**K5. Traditional Healer:**

Did you go to a traditional healer, traditional doctor, witch doctor Yes

or bone setter for this problem? No

**K6. Disability:**

| Problem 1 | Problem 2 | Problem 3 |
| --- | --- | --- |
|  |  |  |
|  |  |  |
|  |  |  |
|  |  |  |
|  |  |  |

Does this problem still impact your daily life?

The condition is not disabling

I feel ashamed

I'm not able to work like I used to

I need help with transportation

I need help with daily living

*[Disability: a physical problem that impacts your life, or makes it difficult to carry out your daily activities. Select the highest level of disability the person experiences. Select only one answer.]*

*[Ask if the person had another problem in this anatomical location. If so, go to the following column (problem 2 or 3) to register this problem. If there are no other problems in this location, go to the following anatomical location. In case of more than 3 problems, mark the ones which are most recent and most relevant for the respondent.]*

L. BREAST

*[only ask the questions in this section if the participant is a female.]*

**L1. Breast:**

Have you ever had a wound, burn, infection that needed to be drained or opened, deformity, breast mass, or an operation on your breast?

___ Yes

___ No

*[If there were/are no problems with this anatomical section you can continue with section H. If the person had a problem with this anatomical section, answer all the questions for problem 1 before moving on to any additional problems.]*

**L2.1. Breast specifics:**

| Problem 1 | Problem 2 | Problem 3 |
| --- | --- | --- |
|  |  |  |
|  |  |  |
|  |  |  |
|  |  |  |
|  |  |  |
|  |  |  |

Tell me what problem you have had.

Wound or infection injury related

Wound or infection not injury related

Burn

Breast cancer

Deformity congenital

Deformity acquired

*[Wound: Open skin; sometimes leaking blood, pus or liquid*

*Deformity: An abnormal tissue arrangement, malformation*

*Congenital: The person is born with the problem.*

*Acquired: The person got the problem later in life.*

**L2.2 Type of injury / accident:**

Did the problem start after an injury or accident? What kind of accident?

| Problem 1 | Problem 2 | Problem 3 |
| --- | --- | --- |
|  |  |  |
|  |  |  |
|  |  |  |
|  |  |  |
|  |  |  |
|  |  |  |
|  |  |  |
|  |  |  |
|  |  |  |
|  |  |  |

*[Pick the one that best describes the injury / accident. Pedestrian and bicycle crash definition: there was no motorized vehicle involved. All can be intentional or unintentional.]*

No, it was not due to an injury / accident

Car, truck, bus crash

Motorcycle crash

Pedestrian, bicycle crash

Gunshot

Stab / slash / cut / crush

Bite or animal attack

Fall

Open fire / explosion

Hot liquid / hot object

| Problem 1 | Problem 2 | Problem 3 |
| --- | --- | --- |
|  |  |  |
|  |  |  |
|  |  |  |

**L3.1. Timing:**

When did this problem start?

In the last month

During the past 12 months but longer than a month ago

Longer than 12 months ago

| Problem 1 | Problem 2 | Problem 3 |
| --- | --- | --- |
|  |  |  |
|  |  |  |

**L3.2 At this moment:**

Do you have this problem now (or during the last week)? Yes No

| Problem 1 | Problem 2 | Problem 3 |
| --- | --- | --- |
|  |  |  |
|  |  |  |

**L4.1 Healthcare sought:**

Did you go to a health facility or see a doctor/nurse for this problem? Yes No

**L4.2 Type of healthcare received:** *[Only when ‘yes’ to L4.1]*

What kind of treatment did you receive?

*[A major procedure is a procedure which requires regional/spinal/general anesthesia; A minor procedures is a procedure requiring local anaesthesia, dressings, wound care, punctures, suturing or incision and drainage]*

| Problem 1 | Problem 2 | Problem 3 |
| --- | --- | --- |
|  |  |  |
|  |  |  |
|  |  |  |

None / No surgical care

Major procedure

Minor procedures

**L4.3 Reason for not having surgical care:** *[Only when ‘none / no surgical care’ to L4.2 or ‘no’ to L4.1]*

What was the main reason not to go to a health facility to see a doctor/nurse or not to have an operation or dressings?

*[Select all the answers that are true. If the person was referred but did not go to the referral hospital, mark the answer why (s)he did not go here.]*

| Problem 1 | Problem 2 | Problem 3 |
| --- | --- | --- |
|  |  |  |
|  |  |  |
|  |  |  |
|  |  |  |
|  |  |  |
|  |  |  |

No money for health care

No (money for) transportation

No time

Fear / no trust

Not available (facility/personnel/equipment)

No need

| Problem 1 | Problem 2 | Problem 3 |
| --- | --- | --- |
|  |  |  |
|  |  |  |

**L5. Traditional Healer:**

Did you go to a traditional healer, traditional doctor, witch doctor Yes

or bone setter for this problem? No

**L6. Disability:**

| Problem 1 | Problem 2 | Problem 3 |
| --- | --- | --- |
|  |  |  |
|  |  |  |
|  |  |  |
|  |  |  |
|  |  |  |

Does this problem still impact your daily life?

The condition is not disabling

I feel ashamed

I'm not able to work like I used to

I need help with transportation

I need help with daily living

*[Disability: a physical problem that impacts your life, or makes it difficult to carry out your daily activities. Select the highest level of disability the person experiences. Select only one answer.]*

*[Ask if the person had another problem in this anatomical location. If so, go to the following column (problem 2 or 3) to register this problem. If there are no other problems in this location, go to the following anatomical location. In case of more than 3 problems, mark the ones which are most recent and most relevant for the respondent.]*

M. WOMEN’S HEALTH

**M1. Reproductive age screening:**

Have you had a bleeding cycle in the last year?

___ Yes

___ No

___ Male

___ Girl under the age of 12 years

*[If ‘no’, skip to question M19.1 about family planning. If ‘male’ or ‘girl under the age of 12 years’ you can skip all the following questions and go to the end of this form to finish the survey with a last check of the survey and your signature.]*

**M2. Gravida:**

How many times, including miscarriages and any current pregnancy, have you been pregnant?

_____

*[If the answer is 0, continue to question M11]*

**M3.1 Pregnant:**

Are you currently pregnant?

___ Yes

___ No

___ I don’t know

**M3.2 Bleeding during pregnancy:** *[Only for those who are currently pregnant.]*

Have you had bleeding during your current pregnancy?

___ Yes

___ No

**M3.3 Gestational Age:** *[Only for those who are currently pregnant.]*

How many months are you pregnant?

_____

**M4. Parity:** *[THIS CANNOT BE MORE THAN THE ANSWER TO QUESTION M2]*

How many times have you delivered a baby? *[Includes C-sections and instrumental deliveries]*

_____

*[If the answer is 0, continue with question M11]*

**M5. Home deliveries:** *[THIS CANNOT BE MORE THAN THE ANSWER TO QUESTION M4]*

How many babies were delivered at home?

_____

**M6. Health facility deliveries:** *[THIS CAN BE CALCULATED FROM QUESTION M4 MINUS M5]*

How many babies were delivered in a health facility?

_____

**M7. C-section:** *[THIS CANNOT BE MORE THAN THE ANSWER TO QUESTION M6]*

How many times where your babies delivered with an abdominal delivery / Cesarean?

_____

**M8. Instrumental deliveries:** *[THIS CANNOT BE MORE THAN THE ANSWER TO QUESTION M6]*

How many times where your babies delivered with the help of instruments (Vacuum / Forceps)?

_____

**M9.1 Obstructed Labor:**

How many times did the baby take too long in coming or a health care worker told you that a Cesarean/abdominal delivery was necessary but you were not able to obtain a Cesarean/abdominal delivery?

_____

**M9.2 Reason for not having Cesarean:** *[Only respond if answer to M9.1 is >0.]*

What was the main reason not to have a Cesarean?

*[Select all the answers that are true. If the person was referred but did not go to the referral hospital, mark the answer why (s)he did not go here.]*

___ No money for health care

___ No (money for) transportation

___ No time

___ Fear / no trust

___ Not available (facility/personnel/equipment)

___ No Need

**M10. Breastfeeding:**

Are you currently breastfeeding?

___ Yes

___ No

| The following questions are about your menstrual period. |
| --- |

**M11. Length of period:**

How long does your period usually last? (number of days)

_____

**M12. Regularity:**

Does your period come regularly?

___ Yes

___ No

**M13. Intermittent bleeding:**

Do you have small bleedings in-between your period?

___ Yes

___ No

**M14. Pain:**

Do you have pain during your period so that you cannot work?

___ Yes

___ No

**M15. Pads or towels/cloths:**

Do you use pads or towels/cloths?

___ Pads

___ Towels/cloths

**M16. Pads/towels:**

How many sanitary pads/towels do you use on the heaviest day of your period?

_____

**M17.1 Health care needed:**

Is there, in your opinion, need for healthcare for your menstrual cycle (such as too much bleeding or too much pain)?

___ Yes

___ No

**M17.2 Possibilities for health care** *[only if M17.1 is Yes]*

Do you have the possibility for health care for your menstrual problem?

___ No, no money for health care

___ No, no (money for) transportation

___ No, no time

___ No, because of fear / no trust

___ No, not available (facility/personnel/equipment)

___ Yes, will go

**M18. Traditional healer:**

Is there need to go to a traditional healer for your menstrual cycle?

___ Yes

___ No

**M19.1 Family Planning:**

**Do you use a family planning method at the moment?** *[Not including traditional/rhythm methods]*

___ Yes

___ No

**M19.2 Type of family planning?** *[only if M19.1 is answered with ‘Yes’]*

What method do you use currently?

___ Contraceptive pills

___ Implant

___ Injectable

___ Intra uterine device / coil

___ Condom

___ Other (surgical methods)

| ‘Thank you very much for answering these questions. Let me check everything to make sure it is complete.’ |
| --- |

*[Go over all the questions to ensure that you have everything. Ask the questions again questions that you accidentally skipped.*

*I checked the full Survey, there is no data missing:*

*______________ [date] _______________ [name] _______________ [signature of interviewer]*

| I see that I have everything that I need; your survey is complete. Thank you again on behalf of the research team. Do you have questions for me? You are always welcome to contact us. Have a nice day. |
| --- |

*If you wish to make a comment for this interview you can do that below:*

____________________________________________________________________________________________________________________________________________________________________________________________________________________________________________________________________________________________________________________________________________________________________________________________________________________

[TIME: ____ hour ____ min]

(REPEAT Sections E-M for 2^nd^ Respondent)
